# Hybridisation‐based target enrichment of phenology genes to dissect the genetic basis of yield and adaptation in barley

**DOI:** 10.1111/pbi.13029

**Published:** 2018-12-06

**Authors:** Camilla Beate Hill, Tefera Tolera Angessa, Lee‐Anne McFawn, Debbie Wong, Josquin Tibbits, Xiao‐Qi Zhang, Kerrie Forrest, David Moody, Paul Telfer, Sharon Westcott, Dean Diepeveen, Yanhao Xu, Cong Tan, Matthew Hayden, Chengdao Li

**Affiliations:** ^1^ Western Barley Genetics Alliance Western Australian State Agricultural Biotechnology Centre School of Veterinary and Life Sciences Murdoch University Murdoch WA Australia; ^2^ Department of Primary Industries and Regional Development, Agriculture and Food South Perth WA Australia; ^3^ Agriculture Victoria Research AgriBio, Centre for AgriBioscience Bundoora Vic. Australia; ^4^ InterGrain Pty Ltd Roseworthy WA Australia; ^5^ Australian Grain Technologies Pty Ltd (AGT) Roseworthy SA Australia; ^6^ Hubei Collaborative Innovation Centre for Grain Industry Yangtze University Jingzhou Hubei China; ^7^ School of Applied Systems Biology La Trobe University Bundoora Vic. Australia

**Keywords:** association mapping, Hordeum vulgare, flowering time, grain yield, next‐generation sequencing, target capture

## Abstract

Barley (*Hordeum vulgare* L.) is a major cereal grain widely used for livestock feed, brewing malts and human food. Grain yield is the most important breeding target for genetic improvement and largely depends on optimal timing of flowering. Little is known about the allelic diversity of genes that underlie flowering time in domesticated barley, the genetic changes that have occurred during breeding, and their impact on yield and adaptation. Here, we report a comprehensive genomic assessment of a worldwide collection of 895 barley accessions based on the targeted resequencing of phenology genes. A versatile target‐capture method was used to detect genome‐wide polymorphisms in a panel of 174 flowering time‐related genes, chosen based on prior knowledge from barley, rice and *Arabidopsis thaliana*. Association studies identified novel polymorphisms that accounted for observed phenotypic variation in phenology and grain yield, and explained improvements in adaptation as a result of historical breeding of Australian barley cultivars. We found that 50% of genetic variants associated with grain yield, and 67% of the plant height variation was also associated with phenology. The precise identification of favourable alleles provides a genomic basis to improve barley yield traits and to enhance adaptation for specific production areas.

## Introduction

Barley (*Hordeum vulgare* L.) is one of the earliest domesticated crops found at Neolithic farming sites in the Fertile Crescent of the Eastern Mediterranean region (Abbo *et al*., 2010). Since its initial domestication, barley has been adapted across a range of diverse agricultural environments and is now cultivated on 46.9 million hectares of land in more than 100 countries worldwide (FAOSTAT 2016, http://www.fao.org/faostat). Grain yield is the most important driver for grower profitability and is thus a key breeding target for genetic improvement. Yield is however a challenging breeding target as it is controlled by a large number of genes with varying effects, and also strongly influenced by genotype by environment interactions (complex trait) (Araus *et al*., 2008). Furthermore, barley has a large and complex genome, similar to other *Triticae* crops such as wheat, making genome‐based selection difficult.

Crop biomass and grain yield depend on a plant's ability to efficiently capture and utilise available resources to produce dry matter, and to avoid or tolerate a wide range of abiotic and biotic constraints. In barley, phenology genes that determine heading date and photoperiod sensitivity drive adaptation to different geographic environments and cropping systems (Andres and Coupland, 2012; Russell *et al*., 2016). Extensive research in the model plant *Arabidopsis* has revealed many of the mechanisms that control flowering (Blümel *et al*., 2015; Hill and Li, 2016). However, little is known about how natural genetic variation within phenology genes impacts grain yield and adaptation in barley.

Genomics‐based strategies for crop improvement, such as genome‐wide association studies (GWAS), can be used to dissect the genetic basis for variation in the expression of complex traits. GWAS exploits the linkage disequilibrium (LD) present in a diverse population of distantly related or unrelated individuals, including natural populations or germplasm collections (Myles *et al*., 2009). Germplasm collections generally contain a broader gene pool with more genetic diversity than segregating progenies. Since association mapping exploits all the recombination events that have occurred in the evolutionary history of the GWAS population, markers significantly associated with a phenotype are typically much closer to the causal variant than those in a segregating population with a limited history of recombination.

In this study, we conducted a comprehensive genomic assessment of a worldwide collection of 895 domesticated barley accessions based on the targeted resequencing of 174 phenology‐related genes and gene homologs. We used target capture based on in‐solution hybridization, a method that focuses only a subset of the genome and thus significantly reduces the sequencing space and cost. Identified genetic variation was used to conduct genome‐wide association analyses to identify key genes linked with phenology and grain yield traits in current barley breeding germplasms. Our results were used to pinpoint key genes that have changed over time due to breeding, starting from the barley cultivars first introduced into Australia to the most recently released varieties. The current results provide critical information for genome‐wide selection to improve yield and adaptation. Diagnostic markers developed in this study to capture the range of genetic variation for phenological adaptation genes in barley will facilitate future breeding of higher yielding cultivars.

## Results

### Targeted re‐sequencing of phenology genes

Targeted re‐sequencing of 174 barley phenology genes (Data S1) were run across 952 domesticated, landrace and breeding accessions from 41 countries. Eighty‐six percent of the accessions represented breeding germplasm from Australian, North American and European programmes (Figure S1a). To maximise phenotypic differences in phenology and grain yield, accessions representing six and two row types, spring and winter growth habit, as well as malting and feed‐end‐use were selected, with an emphasis on spring‐type (92%) and two‐row varieties (90%) (Figure S1b), these being most relevant to the major barley production areas in Australia, Europe and North America. A table of the germplasm in the core panel is provided as Data S2. The resulting sequence dataset consisted of 1.12 billion 150‐bp paired‐end reads (203 Gbp of raw data). After quality filtering the sequence data, 895 barley genotypes were retained and used for alignment, variant discovery and genotype calling.

### Genomic diversity across barley accessions

The SNP discovery pipeline identified a total of 467 339 SNPs, of which 29 822 were within gene‐coding regions, including 6030 SNPs which were captured within the targeted phenology gene regions. On a per sample basis, about 75% of the SNP detected within the captured regions had a mean coverage depth of 25 reads per SNP. As expected for an inbred cereal crop, the rate of heterozygosity for each variety was low (approximately 2% on average). In 170 out of the 174 targeted candidate genes regulating phenology in barley we identified regulatory (3′‐UTR/5′‐UTR/intron/upstream/downstream) as well as coding (frameshift/splice variant/missense/synonymous) SNP variants (Data S3). Approximately 26% and 34% of all SNPs fell within exonic and intronic sequences respectively, based on the predictions of the Variant Effect predictor (VEP) tool (McLaren *et al*., 2016). Of the 6030 SNPs that where within the targeted phenology gene regions, 2350 SNPs had more than 10% missing data, and 1109 SNPs had a MAF of less than 0.01. After filtering and pruning, a total of 2758 SNPs were retained. Additional filtered 1502 SNPs that where outside the targeted phenology gene regions were added to the retained 2758 SNPs to create the final set of 4260 high‐quality filtered SNPs (Data S4, Figure S2a). After pruning and filtering, no systematic bias was present for the minor allele frequency (MAF) of all variants and confirmed expectations of a high proportion of variants with a low MAF and a rapid decline in the proportion of variants with higher MAFs (Figure S2b). From the SNP data, sequence diversity (Polymorphism Information Content, PIC) was estimated at 0.21 across accessions (Table 1).

**Table 1:** Summary of molecular diversity and polymorphism information content for the whole panel and all the subgroups

Group	Average MAF	No. genotypes	PIC
Whole panel	0.18	895	0.21
2‐rowed group	0.18	810	0.2
6‐rowed group	0.22	81	0.24
Spring	0.18	743	0.2
Winter/facultative	0.2	69	0.22
Australian	0.17	328	0.19
International	0.19	569	0.21

MAF, Minor allele frequency; PIC, Polymorphism information content.

Observed mean PIC values are slightly higher for the six‐rowed group (0.24) than for two‐rowed barleys (0.2), for winter (0.22) than for spring barleys (0.2), and for non‐Australian (0.21) than for Australian (0.19) varieties.

**Figure 1:**
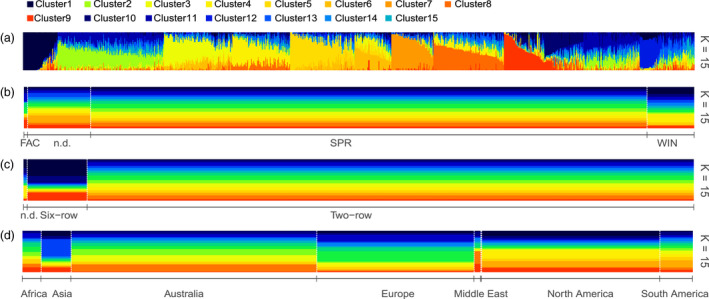
Plot of ancestry estimates inferred by ADMIXTURE for 895 worldwide barley accessions for 4260 SNPs. (a) Each colour represents a population, and the colour of individual haplotypes represents their proportional membership in the different populations. Distribution of ADMIXTURE‐defined populations based on (b) three growth habits, (c) two row types, and (d) seven geographical locations. FAC, facultative; SPR, spring; WIN, winter growth habit.

Underlying population structure is a recognised source of error and needs to be accounted for in GWAS (Myles *et al*., 2009). Known sources of population structure in domesticated barley varieties includes the separation of two‐row and six‐row germplasm pools, which occurred early in barley domestication (Malysheva‐ Otto *et al*., 2006; Pasam *et al*., 2012). Also, within spring barley the loss of a vernalisation requirement has created population structure based on growth habit (Figure 1). Investigation of population structures of the worldwide barley germplasm collection in this study using ADMIXTURE (Alexander and Lange, 2011) to select the optimal K predicted that the optimal number of subpopulations was approximately *K* = 15 (Figures S3 and S4).

A neighbour‐joining (NJ) tree based on genetic distances for the entire population (Figure S5), and a selection of 82 leading Australian cultivars (Figure S6) was constructed. Principal component Analysis (PCA) was performed with separation based on row‐type, growth habit, or geographic location (Figure S7a–c).

Pairwise LD analysis using the entire panel and 4260 SNPs showed LD to gradually decrease with physical distance (Figure S8). The intra‐chromosomal mean *r* ^2^ of 0.068 of the entire panel was much lower compared to studies using markers distributed across the entire genome in a spring barley collection (Pasam *et al*., 2012) as a result of bias caused by most SNPs being confined to a defined capture space (Table S1). While wild barley has remarkably low levels of LD for a self‐pollinating plant (Morrell *et al*., 2005), the extent of LD in cultivated barley as used in this study is greater as a result of self‐fertilisation and relatively small effective population size and is in agreement with previous findings (Caldwell *et al*., 2006; Hamblin *et al*., 2011). Long‐range LD is suggestive of admixture in this population. LD plots for each chromosome are provided in Figure S9.

### Association analysis

Barley accessions were grown in multi‐environment field trials, conducted over five diverse geographical locations across two years in Western Australia (Table S2). Each trial contained between ~50% and 90% of the original set of 895 barley varieties to maximise the number of accessions evaluated. GWAS were performed for three traits; days to Z49 (Zadoks *et al*., 1974) (Zadoks stage 49) as an equivalent for flowering time (Alqudah and Schnurbusch, 2017) grain yield, and plant height, using the 4260 high‐quantity filtered SNPs (Table S3). Manhattan plots and quantile‐quantile plots of the three traits are shown with the results from both the naive model and the MLM model incorporating Q and K in Figures S10–S25.

By using the empirical threshold, we identified 3230 individual significant marker trait associations for the three traits across the twelve environments (Data S5). Of these, 988 unique association signals were detected for flowering time (days to Z49) across 136 genes, of which 543 signals (*q*FDR < 0.05) within 95 genes were detected in twelve environments (Figures S10–S21). At *q*FDR < 0.01, 27 markers were associated with phenology in all twelve environments, with 25 markers located within five target genes (*ADA2*, HORVU5Hr1G095400; *AGLG1*, HORVU5Hr1G095710; *VRN‐H1*, HORVU5Hr1G09563; *CK2A*, HORVU5Hr1G097230; and *PhyC,* HORVU5Hr1G095530). Five markers were located outside of the target gene regions, one of which was captured in the downstream region of *AGLG1*. Association signals of these markers were mostly present on chromosome 5H, with marker *R* ^2^ ranging from 1.2% for Chr_5_603618535 to 13.12% for Chr_Un_77793106 (Table 2).

**Table 2:** Loci and SNPs significantly associated with phenology in all 12 environments

SNP ID^a^	Alleles^b^	MAF	*R* ^2^ (%)^c^	*q* value^d^	Candidate gene^e^	Annotation^f^
Chr_5_598231895	A:G	0.13	3.1–6.8	2.00E‐03	HORVU5Hr1G095400	ADA2
Chr_5_598560301	T:C	0.04	4.8–10.8	6.10E‐04	HORVU5Hr1G095530	PhyC
Chr_5_599019952	T:C	0.04	3.6–12.5	7.30E‐05	n.d.	n.d.
Chr_5_599069784	A:G	0.04	2.5–10.8	7.20E‐04	n.d.	n.d.
Chr_5_599112501	T:C	0.04	3.2–10.2	7.20E‐04	HORVU5Hr1G095630	VRN‐H1
Chr_5_599329482	A:T	0.04	2.6–11.9	3.40E‐03	n.d.	n.d.
Chr_5_599331920	C:T	0.04	3.3–10.5	3.60E‐03	HORVU5Hr1G095710	AGLG1
Chr_5_599332514	T:C	0.14	1.6–6.3	1.00E‐02	HORVU5Hr1G095710	AGLG1
Chr_5_599332925	C:T	0.04	2.8–10.5	1.10E‐03	HORVU5Hr1G095710	AGLG1
Chr_5_599333006	C:T	0.04	2.7–10.2	1.80E‐03	HORVU5Hr1G095710	AGLG1
Chr_5_603612743	G:A	0.03	2.7–6.4	3.50E‐03	HORVU5Hr1G097230	CK2A
Chr_5_603613468	T:G	0.03	2.3–6.4	3.10E‐03	HORVU5Hr1G097230	CK2A
Chr_5_603613940	A:C	0.03	2.7–6.4	2.00E‐03	HORVU5Hr1G097230	CK2A
Chr_5_603614147	A:G	0.03	2.7–6.4	3.50E‐03	HORVU5Hr1G097230	CK2A
Chr_5_603614684	A:G	0.03	2.7–6.4	2.40E‐03	HORVU5Hr1G097230	CK2A
Chr_5_603614823	A:G	0.03	2.7–6.7	2.90E‐03	HORVU5Hr1G097230	CK2A
Chr_5_603614892	A:G	0.04	2.7–6.4	2.00E‐03	HORVU5Hr1G097230	CK2A
Chr_5_603615644	G:A	0.03	3.3–6.6	1.80E‐03	HORVU5Hr1G097230	CK2A
Chr_5_603615648	T:C	0.03	3.3–6.6	1.80E‐03	HORVU5Hr1G097230	CK2A
Chr_5_603615849	G:A	0.04	2.2–5.5	2.50E‐03	HORVU5Hr1G097230	CK2A
Chr_5_603616133	G:C	0.03	2.6–6.4	1.90E‐03	HORVU5Hr1G097230	CK2A
Chr_5_603616317	G:A	0.04	2.7–6.5	2.00E‐03	HORVU5Hr1G097230	CK2A
Chr_5_603616476	T:C	0.03	1.4–6.4	3.60E‐03	HORVU5Hr1G097230	CK2A
Chr_5_603618535	C:T	0.04	1.2–7	2.30E‐03	HORVU5Hr1G097230	CK2A
Chr_5_603618541	C:T	0.05	1.7–6.8	3.70E‐03	HORVU5Hr1G097230	CK2A
Chr_5_646893378	A:C	0.03	2.3–6.5	2.20E‐03	n.d.	n.d.
Chr_Un_77793106	T:C	0.04	5.5–13.1	7.40E‐06	n.d.	n.d.

MAF: Minor allele frequency. *R* ^2^: Contribution to phenotypic variation. *q*FDR < 0.01, for full list (*q*FDR < 0.05) see Data S5.

a Starts with chromosome number followed by physical location of the markers on that chromosome.

b Respect to minor allele.

c Minimum and maximum value of 12 environments.

d Average of 12 environments; *q*‐value = FDR adjusted *P*‐value, significant at *q *<* *0.05.

e Genes annotated in barley genome assembly IBSC v2 or based on BLAST homology to known candidate genes in other plant species were used as the source of candidate genes.

f For details see Data S2.

For grain yield, 148 unique association signals (*q*FDR < 0.05) located within 34 target genes in four environments were identified (Data S5, Figures S22–S25). About 80% of these marker‐trait associations were detected in a single environment, while 30 markers were associated with grain yield in two or more environments. Twenty‐two markers were located within the coding regions of sixteen genes (HORVU1Hr1G011030, HORVU6Hr1G002330, HORVU5Hr1G095710, HORVU5Hr1G095630, HORVU2Hr1G063800, HORVU5Hr1G080430, HORVU5Hr1G080420, HORVU5Hr1G080450, HORVU2Hr1G041090, HORVU5Hr1G080230, HORVU3Hr1G027460, HORVU3Hr1G035680, HORVU3Hr1G090980, HORVU5Hr1G095530, HORVU3Hr1G091000 and HORVU5Hr1G081620) on chromosomes 1H, 2H, 3H, 5H and 6H. Eight markers were captured outside of the target gene regions: Three were located 27–36 bp downstream of HORVU5Hr1G080430, and the other two were present in the promoter regions of HORVU5Hr1G080310 and HORVU5Hr1G095710 respectively (Table 3).

**Table 3:** Loci and SNPs significantly associated with grain yield in at least two environments

SNP ID^a^	Alleles	MAF^b^	*R* ^2^ (min–max)^c^	*q* value^d^	Candidate gene^e^	Annotation^f^
Chr_1_26220293	T:C	0.08	2.4–3.9	6.30E‐03	HORVU1Hr1G011030	n.d.
Chr_2_201118611	G:A	0.32	3.9–5	4.10E‐03	HORVU2Hr1G041090	CBF8A
Chr_2_432062326	G:A	0.31	3.7–4.1	4.30E‐03	HORVU2Hr1G063800	BM8
Chr_3_117876749	T:C	0.35	2.7–2.9	2.60E‐02	HORVU3Hr1G027460	CKX
Chr_3_198828628	T:C	0.16	2.1–3.1	2.20E‐02	HORVU3Hr1G035680	FPF1
Chr_3_634080004	T:C	0.09	2.8–2.9	2.60E‐02	HORVU3Hr1G090980	GA20ox2
Chr_3_634189000	A:G	0.1	2.9–3.3	2.30E‐02	HORVU3Hr1G091000	PI‐2
Chr_3_634190769	T:C	0.1	2.8–3.2	2.30E‐02	HORVU3Hr1G091000	PI‐2
Chr_5_559462764	C:T	0.12	3.7–4	5.50E‐03	HORVU5Hr1G080230	CBF9
Chr_5_559687817	C:T	0.11	5.2–23.5	2.20E‐03	n.d.	n.d.
Chr_5_560571147	T:A	0.11	3.3–3.8	3.60E‐03	HORVU5Hr1G080420	CBF3
Chr_5_560588191	C:T	0.1	3.9–5.2	2.20E‐03	HORVU5Hr1G080430	CBF10A
Chr_5_560588206	A:T	0.1	4.2–4.6	3.10E‐03	HORVU5Hr1G080430	CBF10A
Chr_5_560588247	A:G	0.49	3.2–3.4	2.10E‐02	n.d.	n.d.
Chr_5_560588251	T:G	0.49	3.2–3.5	2.30E‐02	n.d.	n.d.
Chr_5_560588256	C:A	0.09	4.5–5.2	2.20E‐03	n.d.	n.d.
Chr_5_560732040	T:C	0.12	3–4.1	3.50E‐03	HORVU5Hr1G080450	CBF6
Chr_5_560732097	C:G	0.12	3.2–4.5	3.10E‐03	HORVU5Hr1G080450	CBF6
Chr_5_565156748	C:G	0.16	2.6–3	2.60E‐02	HORVU5Hr1G081620	PRR95
Chr_5_565157545	G:C	0.16	2.8–3.2	2.60E‐02	HORVU5Hr1G081620	PRR95
Chr_5_598560301	T:C	0.04	3.1–4.1	3.10E‐03	HORVU5Hr1G095530	PhyC
Chr_5_599019952	T:C	0.04	2.9–4.4	3.30E‐03	n.d.	n.d.
Chr_5_599069784	A:G	0.04	2.9–4.4	3.10E‐03	n.d.	n.d.
Chr_5_599112501	T:C	0.04	2.7–4	4.10E‐03	HORVU5Hr1G095630	VRN‐H1
Chr_5_599329482	A:T	0.04	4.1–5.7	9.40E‐03	n.d.	n.d.
Chr_5_599331920	C:T	0.04	3.6–4.3	2.90E‐03	HORVU5Hr1G095710	AGLG1
Chr_5_599332925	C:T	0.04	2.9–4	3.30E‐03	HORVU5Hr1G095710	AGLG1
Chr_5_599333006	C:T	0.04	2.8–4	2.20E‐03	HORVU5Hr1G095710	AGLG1
Chr_6_6268049	G:A	0.03	2.8–3.1	2.60E‐02	HORVU6Hr1G002330	AGL1
Chr_Un_77793106	T:C	0.04	4.1–5.1	2.20E‐03	n.d.	n.d.

*q*FDR < 0.05. MAF: Minor allele frequency. *R* ^2^: Contribution to phenotypic variation.

a Starts with chromosome number followed by physical location of the markers on that chromosome.

b Respect to minor allele.

c Minimum and maximum value of 2 or 3 environments.

d Average of 2 or 3 environments; *q*‐value = FDR adjusted *P*‐value, significant at *q *<* *0.05.

e Genes annotated in barley genome assembly IBSC v2 or based on BLAST homology to known candidate genes in other plant species were used as the source of candidate genes.

f For details see Data S2.

The marker *R* ^2^ for grain yield ranged from 2.4% for Chr_3_198828628 to 23.5% for Chr_5_559687817. Two of the strongest marker‐trait associations were found for *CBF10A* (HORVU5Hr1G080430), and further five were located within other genes of the CBF gene family (*CBF3*, HORVU5Hr1G080420; *CBF6*, HORVU5Hr1G080450; *CBF8a*, HORVU2Hr1G041090, *CBF9*, HORVU5Hr1G080230), which are known to activate cold or dehydration stress‐inducible genes (Soltész *et al*., 2013). These new loci identified here are attractive candidates for follow‐up studies that could further our understanding of the genetic architecture of these traits.

Of the 148 unique association signals located within 34 target genes that were identified for grain yield, 72 were also significantly associated with phenology (ca. 50%) and located within 22 genes mostly on chromosomes 1H, 3H and 5H, including the following genes: *AGLG1* (HORVU5Hr1G095710), *VRN‐H1* (HORVU5Hr1G095630), *PhyC* (HORVU5Hr1G095530), *CBF10A* (HORVU5Hr1G080430), *CBF3* (HORVU5Hr1G080420), *CBF6* (HORVU5Hr1G080450), *CBF8A* (HORVU2Hr1G041090), *CBF9* (HORVU5Hr1G080230), *CO8* (HORVU7Hr1G027560), *GA20ox2* (HORVU3Hr1G090980), and *PRR95* (HORVU5Hr1G081620; Data S5). Fifteen markers were discovered outside of the target gene space, one of which was detected within the downstream region of *AGLG1* (Chr_5_599329482).

**Figure 2:**
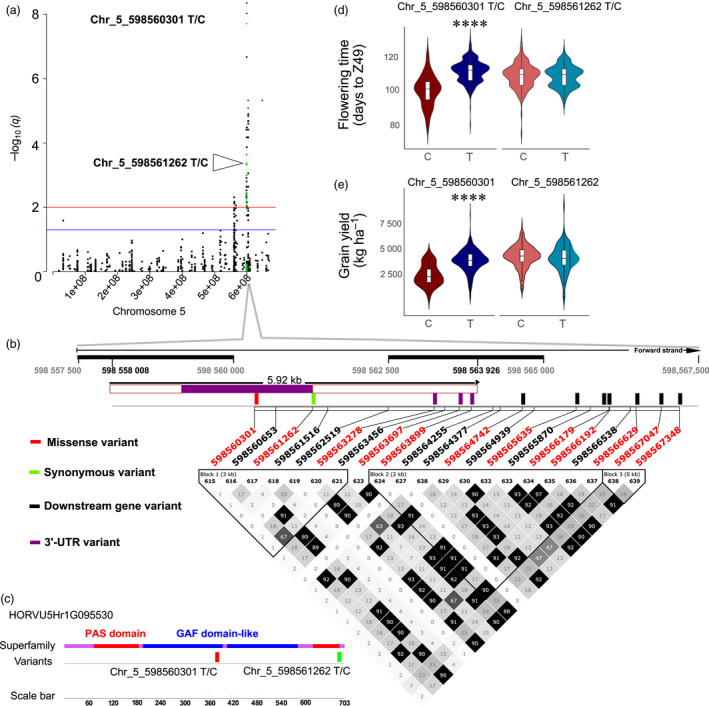
*PhyC* genomic region shows strong association signals for phenology and grain yield. (a) Manhattan plot for chromosome 5H with association signals for phenology (days to Z49, *n* = 462) highlighted in green. GWAS results are presented by negative log_10_ of FDR adjusted *P*‐values (*q*‐values) against position on the chromosomes (*n* = 462). Horizontal dashed lines indicate the genome‐wide significant threshold selected by local false discovery rate and a *q*‐value cut‐off at 0.05 (blue) and 0.01 (red). (b) Summary of local LD and haplotype blocks for the *PhyC* genomic region containing all 21 detected SNPs. LD plot, generated in Haploview, indicates *r* ^2^ values between pairs of SNPs multiplied by 100; white, *r* ^2 ^= 0; shades of grey, 0 < *r* ^2 ^< 1; black, *r* ^2* *^= 1. Haplotype blocks (blocks 1–3) in the *PhyC* genomic region were defined with the four‐gamete rule method. The twelve SNPs that were highly significantly associated with days to Z49 in the optimal MLM are highlighted in red font. (c) The diagrammatic structure of the conserved domains on exon 1 of *PHYC* and location of two variants detected within this region. (d) Days to Z49 variation between different genotypes for Chr_5_598560301_T/C, and Chr_5_598561262_T/C. (e) Grain yield variation between different genotypes for Chr_5_598560301_T/C, and Chr_5_598561262_T/C. *P*‐values calculated using Kruskal‐Wallis tests. ****P value <0.0001.

Twelve SNPs located within HORVU5Hr1G095530 (*PHYC*) were significantly associated with phenology in at least five environments, and one SNP (Chr_5_598560301) in two environments for grain yield (Figure 2). Seven of these SNPs are located downstream of the coding sequence (CDS), three within the 3‐UTR region, and two are present within exon 1 of *PHYC*. One of the SNPs (Chr_5_598560301) is a missense variant located within the GAF‐domain like domain, and the second SNP (Chr_5_598561262), a synonymous variant, is located downstream of the PAF domain (Figure 2c). SNPs in weak linkage disequilibrium were detected nearby, thus suggesting the existence of allelic heterogeneity. For these loci, *R* ^2^ was ranging from 4.8% to 10.8% (Chr_5_598560301), and from 0.7% to 5.8% (Chr_5_598561262) for phenology, and for grain yield, *R* ^2^ was ranging from 3.1% to 4.1% (Chr_5_598560301), and from 1% to <0.1% (Chr_5_598561262) across different environments. Classification of the barley core collection based on the genotype at the Chr_5_598560301 locus shows 11 days earlier flowering when the alternative allele C is present (Figure 2d), which is also significantly associated with lower grain yield (−1344 kg/ha; Figure 2e). This strong effect does not exist between subgroups classified based on different alleles at the locus of the synonymous variant within exon 1 of *PHYC* (Figure 2d,e).

Two hundred sixty unique markers were associated with plant height in five environments (*q*FDR < 0.05) and were mostly localised on chromosomes 3H, and 5H (Data S5, Figures S26–S30). The vast majority were detected in only one environment (ca. 95%), and eleven markers, located within the sequence of seven target genes (HORVU5Hr1G095710, HORVU5Hr1G080420, HORVU3Hr1G091250, HORVU3Hr1G027460, HORVU2Hr1G096300, HORVU3Hr1G027590 and HORVU3Hr1G090980) were present in at least two environments (Table 4).

**Table 4:** Loci and SNPs significantly associated with plant height in at least two environments

SNP ID^a^	Alleles	MAF^b^	*R* ^2^ (min–max)^c^	*q* value^d^	Candidate gene^e^	Annotation^f^
Chr_1_418961516	G:T	0.04	1.6–3.3	3.40E‐02	HORVU1Hr1G057290	n.d.
Chr_2_673851092	G:A	0.04	1.4–4	4.30E‐02	HORVU2Hr1G096300	FD
Chr_3_117875229	C:T	0.01	2.5–2.8	2.00E‐02	HORVU3Hr1G027460	CKX
Chr_3_119254260	C:T	0.11	2.6–2.8	9.50E‐03	HORVU3Hr1G027590	FT2
Chr_3_119256164	G:A	0.02	2.3–2.7	1.70E‐02	n.d.	n.d.
Chr_3_634079937	C:T	0.04	3.9–4.6	2.60E‐02	HORVU3Hr1G090980	GA20ox2
Chr_3_634933260	A:C	0.33	1.7–2.5	1.70E‐02	HORVU3Hr1G091250	CIGARP‐2
Chr_4_610442574	G:A	0.04	1.5–4.7	1.60E‐02	n.d.	n.d.
Chr_5_560570638	C:G	0.04	2.3–3.4	2.20E‐02	HORVU5Hr1G080420	CBF3
Chr_5_599329482	A:T	0.04	2.7–3.7	2.30E‐02	n.d.	n.d.
Chr_5_599333006	C:T	0.06	1.4–2.8	3.90E‐02	HORVU5Hr1G095710	AGLG1

*q*FDR < 0.05. MAF: Minor allele frequency. *R* ^2^: Contribution to phenotypic variation.

a Starts with chromosome number followed by physical location of the markers on that chromosome.

b Respect to minor allele.

c Minimum and maximum value of 2 environments.

d Average of 2 environments; *q*‐value = FDR adjusted *P*‐value, significant at *q *<* *0.05.

e Genes annotated in barley genome assembly IBSC v2 or based on BLAST homology to known candidate genes in other plant species were used as the source of candidate genes.

f For details see Data S2.

Marker *R* ^2^ ranging from 0.3% for Chr_4_610442574 on 4H to 4.6% for Chr_3_634079937, which is located within *GA20ox2* (HORVU3Hr1G090980) on 3H. Two‐thirds of all unique association signals located within seven target genes identified for plant height were also significantly associated with phenology (174 out of 260, ca. 67%). These were located within 33 genes on chromosomes 3H, 5H and 6H, including four genes of the gibberellin pathway: *GA2ox4* (H HORVU1Hr1G076730), *GA2ox3* (HORVU3Hr1G072810), *GA20ox2* (HORVU3Hr1G090980) and *GA2betadiox7* (HORVU3Hr1G117870).

**Figure 3:**
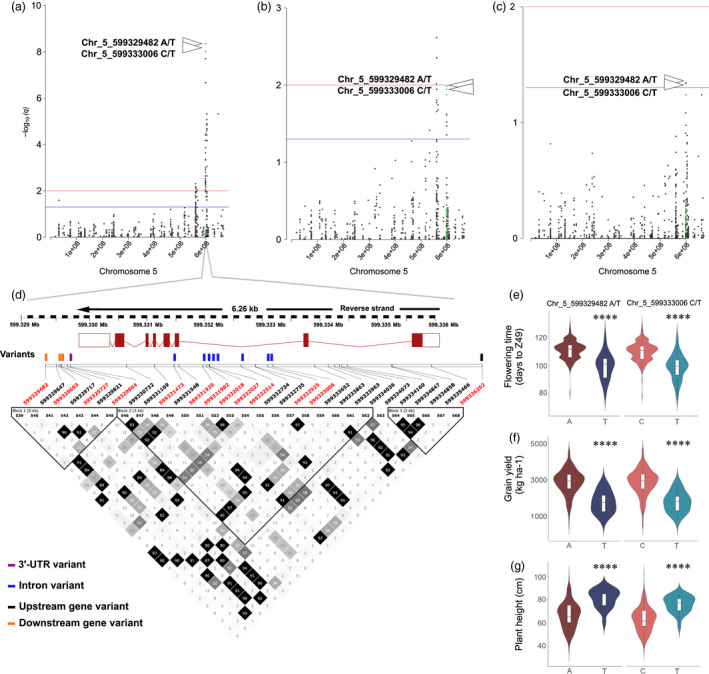
*AGLG1* genomic region shows strong association signals for phenology, grain yield, and plant height. (a) Manhattan plot for chromosome 5H with association signals for flowering time (days to Z49) highlighted in green. (b) Manhattan plot for chromosome 5H with association signals for grain yield (kg/ha) highlighted in green. (c) Manhattan plot for chromosome 5H with association signals for plant height (cm) highlighted in green. All GWAS results are presented by negative log_10_ of FDR adjusted *P*‐values (*q*‐values) against position on each of the seven chromosomes (*n* = 462). Horizontal dashed lines indicate the genome‐wide significant threshold selected by local false discovery rate and a *q*‐value cut‐off at 0.05 (blue) and 0.01 (red). (d) Summary of local LD and haplotype blocks for the *AGLG1* genomic region containing all 21 detected SNPs. LD plot, generated in Haploview, indicates *r* ^2^ values between pairs of SNPs multiplied by 100; white, *r* ^2 ^= 0; shades of grey, 0 < *r* ^2 ^< 1; black, *r* ^2 ^= 1. Haplotype blocks (blocks 1–3) in the *AGLG1* genomic region were defined with the four‐gamete rule method. SNPs that were highly significantly associated with phenology in the optimal MLM are highlighted in red font. (e) Days to Z49 variation between different genotypes for Chr_5_599329482_A/T, and Chr_5_599333006_C/T. (f) Grain yield variation between different genotypes for Chr_5_599329482_A/T, and Chr_5_599333006_C/T. (g) Plant height variation between different genotypes for Chr_5_599329482_A/T, and Chr_5_599333006_C/T. *P*‐values calculated using Kruskal‐Wallis tests. ****P value <0.0001.

Thirteen SNPs located within *AGLG1* (HORVU5Hr1G095710) were significantly associated with phenology (Figure 3). Figure 3a‐c show Manhattan plots of chromosome 5H for all three traits using MLM, and Figure 3d shows the position of all detected variants including those that were significantly associated with phenology, as well as the LD plot surrounding these sites.

Early flowering can be an effective drought escape mechanism but can also limit grain yield potential due to the reduced time available for photosynthetic production and seed nutrient accumulation necessary for higher grain yield (Stratonovitch and Semenov, 2015). In this study, two markers were associated with all three traits (phenology, grain yield and plant height), one located within intron 2 (Chr_5_599333006_C/T), and the other within the downstream region of *AGLG1* (Chr_5_ 599329482_A/T). SNPs in weak linkage disequilibrium were detected nearby, thus suggesting the existence of allelic heterogeneity. For these loci, *R* ^2^ was about 10% (Chr_5_599333006), and 11% (Chr_5_ 599329482) for phenology. For grain yield, *R* ^2^ was ranging from 3% to 4% (Chr_5_599333006), and from 4.1% to 4.8% (Chr_5_ 599329482), and for plant height, *R* ^2^ was ranging from 4.1% to 4.8% (Chr_5_599333006), and from 3% to 3.7% (Chr_5_ 599329482) across different environments. For both variants, the reference allele is the positive allele for phenology and grain yield, and the negative allele for plant height (Data S5). Alleles at these loci separate the 895 accessions into two subgroups, with accessions harbouring the reference allele at these loci to be higher yielding (+1072 to 1102 kg/ha), of shorter stature (−15 cm) and comparably late maturing (+11 days; Figure 3e–g).

### Short growing seasons led to the evolution of earlier flowering onset during breeding in Australia

To understand the impact of breeding on genetic diversity in the barley germplasm collection, we investigated the functional allele diversity detected for significant SNP‐trait associations in different subgroups of the barley phenology core collection: (i) all 328 Australian barley varieties (including advanced breeding lines), (ii) a subset of 82 core commercialized Australian barley cultivars representing Australian barley breeding history of the past 100 years and (iii) all 567 international barley varieties (Data S6). In this study, a functional allele is defined as the allele with the largest absolute effect size for all detected significant marker‐trait associations (qFDR < 0.05).

Based on the GWAS results for phenology, 543 significant SNP‐trait associations were detected in twelve environments. Four hundred and twenty‐nine functional alleles were located within the coding regions of 95 genes, and additional 141 alleles were captured outside of target regions of which some were within regulatory regions up‐or downstream of functional genes. A positive allelic effect, associated with later flowering, was detected for 202 functional alleles, and the remaining 341 were associated with earlier flowering compared to the alternative allele. Up to 32 (HORVU3Hr1G026650, *AG1*), 30 (HORVU5Hr1G097230, *CK2A*) and 29 (HORVU2Hr1G013400, *PPD‐H1*) functional alleles were detected within functional genes. The 328 Australian varieties were polymorphic for 516 out of 543 SNPs, and about 5% (27 alleles within the target region of 15 genes) did not contain allelic diversity, including three SNPs located within *FT2* (HORVU3Hr1G027590) and two SNPs located within HORVU1Hr1G094980 (*ELF3*). Within the subset of 82 key Australian varieties, 50% (or 266 SNPs) were monomorphic and genetically fixed. The monomorphic SNPs were distributed across 61 genes, including HORVU3Hr1G026650 (*AG1*, 27 SNPs), HORVU5Hr1G097230 (*CK2A*, 21 SNPs), HORVU3Hr1G027590 (*FT2*, 15 SNPs), HORVU2Hr1G013400 (*PPD‐H1*, 13 SNPs) and HORVU2Hr1G017290 (*CDF1*, 11 SNPs). Seventy‐four percent of the polymorphic SNPs and 61% of all monomorphic SNPs were associated with earlier flowering, showing that alleles for earlier flowering were gained or retained during breeding in Australian barleys.

Based on the GWAS results for grain yield, 30 SNPs were detected that were significant for two or more environments. Sixteen genes carried 22 functional alleles, and additional eight alleles were captured outside target region. A positive allelic effect, meaning loci were associated with higher grain yield, was detected in approx. 50% of all alleles. Up to three (HORVU5Hr1G095710, *AGLG1*) functional alleles were detected within functional genes. Although within the larger set of Australian barleys, all grain yield‐associated SNPs were polymorphic, 30% were monomorphic within the subset of 82 key Australian varieties. Monomorphic SNPs were distributed across four genes, most monomorphic alleles detected in HORVU5Hr1G095710 (*AGLG1*, 3). All polymorphic and monomorphic SNPs carried a positive allelic effect, showing that alleles for higher grain yield were either acquired or retained during breeding in Australian barleys.

Based on the GWAS results for plant height, 11 SNP were detected that were significant for two or more environments. Eight genes carried one functional allele each, and additional three alleles were captured outside the target region. A negative allelic effect, meaning they are associated with shorter stature, was detected in approx. 80% of all alleles. Within the larger set of Australian barleys, all but one (HORVU3Hr1G027590, *FT2*) SNPs were polymorphic, but approx. 72% were monomorphic within the subset of 82 key Australian varieties. Monomorphic SNPs were distributed across five genes, including in *AGLG1* and *FT2*. The majority of monomorphic SNPs (six out of eight) carried a positive allelic effect, and all polymorphic SNPs carried a negative allelic effect, showing that alleles for shorter plants were acquired in Australian barleys during breeding.

We then traced the change in effect direction of functional alleles during breeding. We focussed on four Australian malting barley varieties representative of key breeding stages in the history of Australian barley breeding: Chevallier, a highly successful British heritage barley variety selected in 1824 (Brookes, 2005), and direct ancestor of Prior (released 1903), the first Australian barley cultivar; Clipper, a key variety of medium maturity that replaced Prior after its release in 1968 from one of the first full‐time breeding programs at the Waite Agricultural Research Institute in South Australia (Friedt, 2011), and La Trobe, a recently released (2014) but widely grown high yielding early maturing variety developed in Australia, and broadly adapted to low and medium rainfall growing areas. La Trobe is a derivative of an elite breeding line from the Victorian breeding program (VB9409) and high yielding feed variety Dash. La Trobe is a reselection from Hindmarsh (released 2006), one of the highest yielding food and feed varieties in Australia, and is also closely related to Prior and Clipper.

Based on the GWAS results for phenology, 41 changes in functional alleles (26 within the target capture region of 18 genes, and 15 outside target capture region), were detected between Chevallier and Prior (Table S4). Genes with the highest number of changes in functional alleles were HORVU1Hr1G076730 (*GA2ox4*, 4), and genes belonging to the photoperiod pathway which were all associated with earlier flowering: HORVU5Hr1G081620 (*PRR95*, 3 loci), and HORVU2Hr1G013400 (*PPD‐H1*, 3 loci). Strikingly, 34 of these changes which distinguished Chevallier from Prior were associated with earlier flowering. Allelic changes that distinguished Prior from Clipper (53; 42 within the target capture region of 14 genes, and 11 outside the target capture region) were concentrated within two genes, *STK* (16, earlier flowering), *PHYC* (11, later flowering), and led to later flowering (32) (Table S5). A comparison between Clipper and La Trobe revealed 66 changes in functional alleles (51 within the target capture region of 15 genes, and 15 outside target capture region) (Table S6). Genes with the highest number of changes in functional alleles, all of which were associated with earlier flowering, were HORVU6Hr1G022330 (*ZTLb*, 11), and HORVU5Hr1G095530 (*PHYC*, 11) which contains the same changes as detected when comparing Prior and Clipper, but in reverse. The vast majority of functional allelic changes (49) were associated with earlier flowering in La Trobe compared to Clipper, and explain the early season phenotype of La Trobe compared to the mid‐season phenotype of Clipper.

Based on the GWAS results for grain yield, three changes in functional alleles were detected between Chevallier and Prior, including two changes in a core clock gene (HORVU5Hr1G081620, *PRR95*), which were associated with lower grain yield (Table S7). Four functional allelic changes within four genes distinguished Prior from Clipper which were all associated with higher grain yield in Clipper (Table S8). Twelve functional allelic changes within six genes distinguished Clipper from La Trobe which were all associated with higher grain yield in La Trobe, including two changes in both *PRR95* (HORVU5Hr1G081620), and HORVU5Hr1G080430 (*CBF10*) (Table S9).

Based on the GWAS results for plant height, no changes in functional alleles were detected between Chevallier and Prior. One functional allelic changes within HORVU3Hr1G090980 (*GA20ox2*) distinguished Prior from Clipper associated with shorter plant stature (Table S9). One functional allelic change within HORVU5Hr1G080420 (*CBF3*) distinguished Clipper from La Trobe associated with taller plants in La Trobe (Table S10).

Genetic markers were developed based on the genetic variants detected in the target enrichment to capture the range of allelic variation for major phenology genes in barley. Sequences of 64 SNPs present in 29 candidate genes were converted into Kompetitive Allele Specific PCR (KASP) assays to detect the specific parental allele for MAS in barley breeding (Data S7).

## Discussion

Second‐generation sequencing technologies allow sequence variation analyses on a genome‐wide scale. However, collecting whole‐genome sequence data on a population scale to a sufficient depth can be still prohibitive, despite rapidly declining sequencing costs. Reduced‐representation sequencing approaches that target only a subset of the genome can be applied to any species for which a draft or complete reference genome sequence is available, allow rapid discovery of thousands of molecular variants, and can be applied for high‐resolution exploration of genetic resources or to dissect quantitative traits (Barbazuk *et al*., 2005). Transcriptome sequencing or exome capture approaches enable targeted identification of sequence variants in protein‐coding genome regions only (Ku *et al*., 2012), whereas target capture approaches based on in‐solution hybridization can identify sequence variants in coding as well as noncoding genome regions.

We applied a target capture approach based on in‐solution hybridization which enabled the rapid and cost‐effective identification of candidate genes associated with phenology and other important agronomic traits in barley. It involves custom design of capture probes targeting specific chromosome regions harbouring quantitative trait loci or candidate genes for traits of interest, which enable extremely flexible scaling of resequencing experiments of few to many genes at low cost in large plant populations. Although the target enrichment data sets may not accurately reflect the allele frequency distributions observed in full resequencing data, and may be skewed toward intermediate and lower frequency alleles, they are very well suited for association mapping of QTL. Using this approach we identified allelic diversity of genes that underlie flowering time, the genetic changes that have occurred during breeding, and their impact on yield within a diverse barley germplasm collection. Most association studies in crop plants have used populations representing global or regional diversity, whereas the population used here focuses on breeding germplasm from Australian, North American and European programmes. While this approach misses some of the diversity present in large worldwide collections (>260 000 accessions (Matus and Hayes, 2002), the advantage is that any favourable alleles identified in this study will already be present in adapted lines, increasing the efficiency of subsequent breeding strategies.

We mapped 988, 148 and 260 unique GWAS signals significantly associated with phenology, grain yield, and plant height respectively, and identified candidate genes for the majority of associated loci. Flowering‐related gene *AGAMOUS‐LIKE GENE1* (*AGLG1*), encoding a grass‐specific SEPALLATA‐like MADS‐box protein (Distelfeld and Dubcovsky, 2010) and *PHYTOCHROME C (PHYC),* encoding the apoprotein of red/far‐red light photoreceptor PHYC (Nishida *et al*., 2013), were identified as major candidates detected for the genomic regions associated with both phenology and grain yield. Alleles in these genes associated with later flowering were also associated with higher grain yield. Peak SNPs at the identified loci explained ~13% of the phenotypic variance for phenology, ~23% of the variance for grain yield, and ~5% of the variance for plant height in the full population. Together with overall high heritability of flowering time scored as days to Z49 in this population, this confirms that the gene capture space contained genes with a good representation that were associated with plant phenology.

Domestication and breeding by humans have had significant impacts upon the frequencies and types of genetic variation that segregate within crop populations. In Australia, early settlers first sowed barley on 3.24 ha shortly after the arrival of the First Fleet at Botany Bay in 1788 (Friedt , 2011). Since then, barley production has expanded rapidly to 1 Mio ha in 1966, and more than 4.6 Mio ha in 2016/2017 (FAOSTAT, 2016). In 2016/17, Australia became the world's top barley exporter, shipping nearly a quarter of all global barley exports by value primarily to Asian countries and the Middle East. Modification of phenology genes was the key element to adapting the introduced late‐maturing and drought‐sensitive European barley varieties, such as Chevalier, to Australian growing conditions (Brookes, 2005). Australia's dryland farming system relies on soil moisture and seasonal rainfall, placing increased emphasis on adaptation to hot and dry environments. In addition, the expansion of barley production into central areas within Western Australia has placed increased emphasis on adaptation to drier environments with a later onset of rainfall during the growing season.

Based on the association analyses results, we examined the past 100 years of barley breeding history in Australia. Among the 30 nucleotide variants associated with grain yield, 15 have been fixed (or close to) in Australian barley varieties over the past 100 years of conventional breeding. Extensive breeding, including the introduction of favourable alleles into elite varieties, have contributed to tripling cereal yields over the past 50 years (Araus and Cairns, 2014). Current Australian cultivars were found to carry a majority of favourable alleles for grain yield in combination with early‐flowering alleles. Analysis of allele diversity in major Australian barley cultivars showed that the selection of varieties with a shorter growth period adapted to late sowing led to substantial yield increases (such as seen in La Trobe and related variety Hindmarsh). Barley cultivars with a shorter growth period can be planted later at a time that coincides with the first winter rainfall. An interesting question is whether this breeding trend for early flowering is also associated with yield stability under a changing global climate with increasing temperature and irregular rainfall. Favourable alleles for grain yield have been gradually enriched during the past 50 years of Australian barley breeding with new varieties containing most of the favourable alleles detected in this study. The result demonstrates the effectiveness of conventional breeding for yield improvement. This study also suggests that the relatively narrow genetic diversity present in the Australian barley gene pool may become a considerable bottleneck for ongoing crop improvement.

Early flowering and maturity can be an effective drought escape mechanism, but was also shown to limit grain yield potential due to the limited time available for photosynthesis, translocation of carbohydrates and total nutrient accumulation to produce high grain yield (Bidinger and Witcombe, 1989; Stratonovitch and Semenov, 2015). In this study, the genotype at the Chr_5_598560301 locus within *PHYC* shows earlier flowering and lower grain yield compared to the alternative allele. Two markers were associated with all three traits (phenology, grain yield and plant height), located within or in the vicinity of *AGLG1*, which also showed associations with earlier flowering and lower grain yield. However, these alleles represent particularly strong effects for both flowering time and grain yield and are not common in Australian germplasm.

Ongoing selection for optimal flowering behaviour with specific regional adaptations will remain critical for the barley grains industry. The ability to fine‐tune flowering to the growing season could give significant advantages for both breeders and growers. Marker‐assisted selection and targeted genetic modification of flowering behaviour combined with faster breeding cycles have great potential to optimise the timing of flowering to specific environments and maximise grain yield (Hill and Li, 2016). Avenues for MAS and targeted genetic modification of flowering behaviour offer the potential to optimise the timing of flowering to specific environments and maximise grain yield, and remains to be explored further. This study shows that targeted enrichment strategy of target genes coupled with GWAS in a diverse population of barley can provide new opportunities to connect sequence diversity to complex phenotypes in crop plants that can be deployed in cultivar improvement through genomic‐assisted breeding.

## Experimental procedures

### Plant material, field experiments and phenotypic data

The barley accessions of the phenology diversity panel were selected from over 4000 accessions preserved at the Western Barley Genetics Alliance at Murdoch University (Perth, Australia) that includes landraces, cultivars and breeding accessions from 41 countries in Europe, Asia, North and South America, Africa and Australia. After preliminary evaluation of heading date and response to photoperiod among the 4000 accessions in field trials in Australia, 952 accessions were selected to represent the diversity for the phenology. This selection represents the entire spectrum of cultivated barley, namely two‐ (90%) and six‐row (10%) genotypes with winter (8%), spring (91%) and facultative (1%) growth habits. The selected germplasm also included all Australian barley varieties released in the past 200 years.

Field experiments were conducted in 2015 and 2016 across multiple diverse environments in Western Australia (South Perth, Geraldton, Merredin, Katanning and Esperance). Geraldton, South Perth and Esperance are located along the coast of the Southern Ocean with high annual rain fall, but have very different daily maximum temperatures (the Geraldton site is the warmest and the Esperance site is the coolest). The distance between Geraldton and Esperance is over 1100 km. The Merredin site is located inland with low rainfall while the Katanning site has medium rainfall. All regional field trials were planted in a randomised complete block design with plots of 1 by 3 m^2^ laid out in a row column format. Field trials at South Perth were conducted using a hill plot technique with 40 cm distance within and between rows. Seven control varieties were used for spatial adjustment of the experimental data. In each plot of each experiment in the study, measurements were taken to determine maturity (days to Z49), plant height and grain yield. Plant maturity was recorded as the number of days from sowing to 50% awn emergence above the flag leaf (Z49) (Zadoks *et al*., 1974) as an equivalent to flowering time (Alqudah and Schnurbusch, 2017). Plant height was determined by estimating the average height of all plants in each plot. Grain yield was determined by destructively harvesting all plant material from each plot to separate the grain to determine grain mass and estimate grain yield (kg/ha). Grain yield data collected from field experiments was analysed using mixed linear model analysis to determine Best Linear Unbiased Predictions (BLUPs) for each trait for further analysis. Local best practices for fertilization and disease control were adopted for each trial site.

### Targeted resequencing of phenology genes

To interrogate the genetic diversity of phenology and phenology‐related genes in a worldwide collection of barley landraces and cultivars, we designed a custom target enrichment sequencing assay that included loci implicated in the flowering pathway in barley and related plant species (Appendix S1).

Genomic DNA was extracted from leaves of a single barley plant per variety using the cetyl‐trimethyl‐ammonium bromide (CTAB) method (Murray and Thompson, 1980). Physical shearing of genomic DNA was performed to an average size of 275 bp using a Covaris S2 Focused‐Ultrasonicator, followed by end repair and A‐tailing of the fragments using unique Illumina 9 nt barcode adapters to generate dual‐indexed libraries. Sample library preparation was conducted with KAPA Hyper Prep Kit (KAPA Biosystems, Wilmington, MA) as per the manufacturer's protocol. Targeted enrichment of genomic DNA regions was performed by solution‐based hybrid capture using a synthetic library consisting of 13 588 probes (MYbaits, MYcroarray^®^, Ann Arbour, MI) following the manufacturer's protocol version 2.3.1. More details can be found in Appendix S1.

### Sequence alignment, variant discovery, genotype calling and variant prediction

Fastq sequence files were post‐run filtered, and reads aligned to the latest release of the barley reference genome assembly (IBSC v2; Mascher *et al*., 2017) using Nuclear software v3.6.16 (GYDLE Inc., Montreal, Canada). SNP variant discovery and genotype calling was performed using custom Perl scripts to produce a VCF version 4.2 genotype file based on the alignment files. Variant effect prediction of SNP captured within gene exon (coding) sequence was performed using the Ensembl Variant Effect predictor toolset (Ensembl Variant Effect Predictor web interface http://www.ensembl.org/vep). More details can be found in Appendix S1.

### Population structure and genotypic data analysis

The model‐based clustering algorithm of ADMIXTURE v.1.3.0 was used to investigate subpopulation structure of the barley diversity panel. The software CLUMPP (Jakobsson and Rosenberg, 2007) v.1.1.2 was used to obtain the optimal alignments of for each K‐value, and plots were made using the cluster visualization program Pophelper v.2.2.3 (http://royfrancis.github.io/pophelper/) implemented in R software (http://www.R-project.org/). Principal component analysis (PCA) was conducted based on all marker data using TASSEL v.5.2.39. More details can be found in Appendix S1.

### Linkage disequilibrium, haplotype and association analysis

Genome‐wide LD analysis was performed among the panel and subgroups by pair wise comparisons among the intra‐chromosomal SNP markers using Haploview (Barrett *et al*., 2005) v.4.2. Genome wide association studies were performed using TASSEL v.5.2.39 software (Bradbury *et al*., 2007). Different statistical models were used to calculate *P*‐values for putative marker‐trait associations which included population structure (Q) and the kinship matrix (K) to account for population structure to avoid spurious associations. Multiple testing was performed to assess the significance of marker trait associations using the qvalue (Storey, 2002) v.2.8.0 R package (R 3.4.2) employing the smoother method (Storey and Tibshirani, 2003), an extension of the false discovery rate (FDR) method. Q‐values were calculated using the R‐package q‐value. The Manhattan plots were drawn with qman (Turner, 2018) v.0.1.4. More details can be found in Appendix S1.

### KASP assay

For KASP assays developed in this study, complete coding sequences were obtained from the latest version of the barley reference genome assembly (IBSC v2) (Data S7). Allele‐specific and common primers for each KASP marker were designed using Geneious^®^ software v. 10.2.3 (Biomatters Ltd., NZ) following standard KASP guidelines. Assays were tested in 384‐well formats and set up as ~6 μL reactions following the manufacturer's instructions (LGC Genomics, Hoddeson, UK). Amplification was carried out on an ABI ViiA7 instrument (Applied Biosystems, Foster City, CA), and end‐point genotyping was performed using the ABI QuantStudio™ Real‐Time PCR software v1.3.

## Availability of data and material

Data generated or analysed during this study are included in this published article (and its supplementary information files). Additional datasets used and/or analysed during the current study are available from the corresponding author on reasonable request.

## Authors’ contributions

CL, TA, DM and PT conceived the project. TA and CL collected the barley accessions, conducted initial phenology evaluation and selected the accessions for this study. TA, LM, SW, YX, XZ and CL conducted the field experiments and phenotyping. DD designed the field trials and conducted the spatial analysis for yield. CH and XZ collected samples and extracted DNA. CH, KF and CT retrieved and prepared the phenology gene sequences for the enrichment probes. CH and DW performed the laboratory work including the DNA library constructions and target enrichments. CH and DW performed the genome data analyses. MH and JT developed the in‐house programmes for the data analyses. SW performed KASP assays. CH designed the KASP assays, and performed the GWAS and all statistical analyses. CH analysed all of the data and wrote the paper with input from CL. CL supervised the project.

## Conflict of interest

DM is affiliated with InterGrain Pty Ltd WA. PT is affiliated with Australian Grain Technologies Pty Ltd (AGT), SA. There are no financial relationships or competing interests on the part of any author that could potentially bias the findings reported in the manuscript.

## Supporting information


**Figure S1.** Phenology diversity panel of core collection barley varieties.
**Figure S2.** Sequence diversity of the barley phenology core collection.
**Figure S3.** Exploration of the optimal number of genetic subpopulations (*K*) using Δ cross‐validation error and standard error values in the barley germplasm collection.
**Figure S4.** Plot of ancestry estimates inferred by ADMIXTURE for 895 worldwide barley accessions for 4260 SNPs.
**Figure S5.** Neighbour‐joining tree of 895 barley varieties.
**Figure S6.** Neighbour‐joining tree of 82 selected Australian barley varieties.
**Figure S7.** Principal component analysis (PCA) of the first two components of 895 barley varieties.
**Figure S8.** The extent of LD in the barley phenology core set of a worldwide collection of domesticated barley varieties.
**Figure S9.** Linkage disequilibrium plots of 4260 SNPs for 895 barley accessions.
**Figure S10.** Manhattan plots of flowering time for the Esperance 2015 environment.
**Figure S11.** Manhattan plots of flowering time for the Esperance 2016 environment.
**Figure S12.** Manhattan plots of flowering time for the Geraldton 2015 environment.
**Figure S13.** Manhattan plots of flowering time for the Geraldton 2016 environment.
**Figure S14.** Manhattan plots of flowering time for the Katanning 2015 environment.
**Figure S15.** Manhattan plots of flowering time for the Katanning 2016 environment.
**Figure S16.** Manhattan plots of flowering time for the Merredin 2016 (non‐irrigated) environment.
**Figure S17.** Manhattan plots of flowering time for the Merredin 2016 (irrigated) environment.
**Figure S18.** Manhattan plots of flowering time for the Perth 2015 (time of planting 1) environment.
**Figure S19.** Manhattan plots of flowering time for the Perth 2015 (time of planting 2) environment.
**Figure S20.** Manhattan plots of flowering time for the Perth 2015 (time of planting 2) environment.
**Figure S21.** Manhattan plots of flowering time for the Perth 2016 environment.
**Figure S22.** Manhattan plots of grain yield for the Esperance 2016 environment.
**Figure S23.** Manhattan plots of grain yield for the Katanning 2015 environment.
**Figure S24.** Manhattan plots of grain yield for the Merredin 2016 (non‐irrigated) environment.
**Figure S25.** Manhattan plots of grain yield for the Merredin 2016 (irrigated) environment.
**Figure S26.** Manhattan plots of plant height for the Geraldton 2015 environment.
**Figure S27.** Manhattan plots of plant height for the Katanning 2015 environment.
**Figure S28.** Manhattan plots of plant height for the Perth 2016 environment.
**Figure S29.** Manhattan plots of plant height for the Merredin 2016 (non‐irrigated) environment.
**Figure S30.** Manhattan plots of plant height for the Merredin 2016 (irrigated) environment.

**Table S1.** The extent of LD in the barley phenology core panel.
**Table S2.** Field site location and climate data during growth period.
**Table S3.** Estimation of mean, minimum (min), maximum (max), median (med), and heritability (*h* ^2^) of all traits.
**Table S4.** Functional allele diversity between Chevallier and Prior based on significant SNP‐associations with phenology.
**Table S5.** Functional allele diversity between Prior and Clipper based on significant SNP‐associations with phenology.
**Table S6.** Functional allele diversity between Clipper and La Trobe based on significant SNP‐associations with phenology.
**Table S7.** Functional allele diversity between Chevallier and Prior based on significant SNP‐associations with grain yield.
**Table S8.** Functional allele diversity between Prior and Clipper based on significant SNP‐associations with grain yield.
**Table S9.** Functional allele diversity between Prior and Clipper based on significant SNP‐associations with plant height.
**Table S10.** Functional allele diversity between Clipper and La Trobe based on significant SNP‐associations with plant height.

**Appendix S1.** Detailed description of additional experimental procedures.

**Data S1.** Information of phenology genes used for targeted re‐sequencing.

**Data S2.** Information of barley phenology core set.

**Data S3.** Variant effect prediction of 6030 SNP captured within the target regions.

**Data S4.** Final filtered SNP set of 4260 SNPs.

**Data S5.** Loci and SNPs significantly associated with flowering time, grain yield and plant height in 12 environments.

**Data S6.** Analysis of functional alleles based on SNPs significantly associated with flowering time, grain yield and plant height.

**Data S7.** Comparison between KASP and target enrichment results.
