# Supplementary material for: Hybridisation‐based target enrichment of phenology genes to dissect the genetic basis of yield and adaptation in barley
Source: Plant Biotechnol J. 2018 Dec 6;17(5):932–44. doi: 10.1111/pbi.13029 (PMC6587706; doi:10.1111/pbi.13029)
Supplement: Supplementary file 1 — Figure S1. Phenology diversity panel of core collection barley varieties. Figure S2. Sequence diversity of the barley phenology core collection. Figure S3. Exploration of the optimal number of genetic subpopulations (K) using Δ cross‐validation error and standard error values in the barley germplasm collection. Figure S4. Plot of ancestry estimates inferred by ADMIXTURE for 895 worldwide barley accessions for 4260 SNPs. Figure S5. Neighbour‐joining tree of 895 barley varieties. Figure S6. Neighbour‐joining tree of 82 selected Australian barley varieties. Figure S7. Principal component analysis (PCA) of the first two components of 895 barley varieties. Figure S8. The extent of LD in the barley phenology core set of a worldwide collection of domesticated barley varieties. Figure S9. Linkage disequilibrium plots of 4260 SNPs for 895 barley accessions. Figure S10. Manhattan plots of flowering time for the Esperance 2015 environment. Figure S11. Manhattan plots of flowering time for the Esperance 2016 environment. Figure S12. Manhattan plots of flowering time for the Geraldton 2015 environment. Figure S13. Manhattan plots of flowering time for the Geraldton 2016 environment. Figure S14. Manhattan plots of flowering time for the Katanning 2015 environment. Figure S15. Manhattan plots of flowering time for the Katanning 2016 environment. Figure S16. Manhattan plots of flowering time for the Merredin 2016 (non‐irrigated) environment. Figure S17. Manhattan plots of flowering time for the Merredin 2016 (irrigated) environment. Figure S18. Manhattan plots of flowering time for the Perth 2015 (time of planting 1) environment. Figure S19. Manhattan plots of flowering time for the Perth 2015 (time of planting 2) environment. Figure S20. Manhattan plots of flowering time for the Perth 2015 (time of planting 2) environment. Figure S21. Manhattan plots of flowering time for the Perth 2016 environment. Figure S22. Manhattan plots of grain yield for the Esperance 2016 environment. Fig [file PBI-17-932-s009.pdf]

a) b)

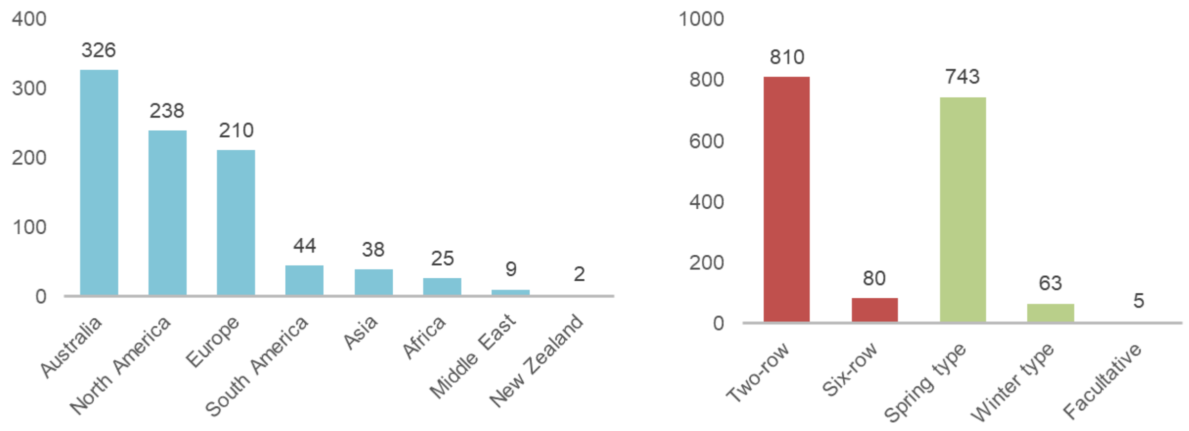

Supplementary Figure S1. **Phenology diversity panel of core collection barley varieties.** a) Geographic origins of barley varieties. Numbers of countries in each geographical region are indicated on top of the bars. b) Row type and growth habit of barley varieties. Numbers of barley varieties are indicated on top of the bars.

a)

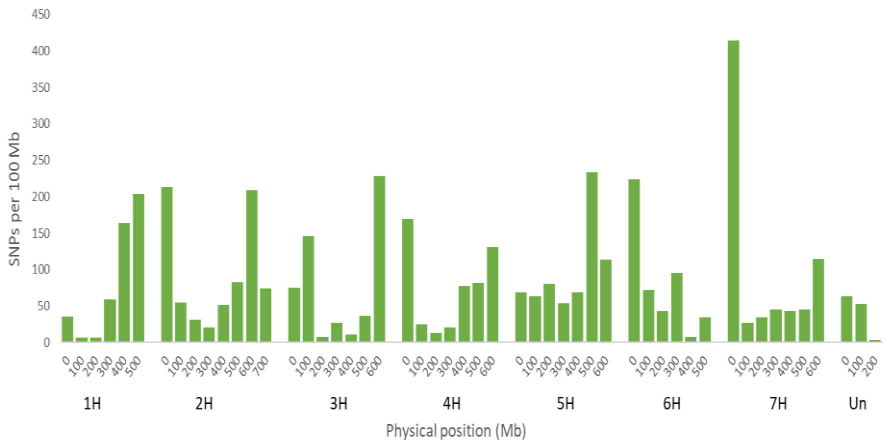

b)

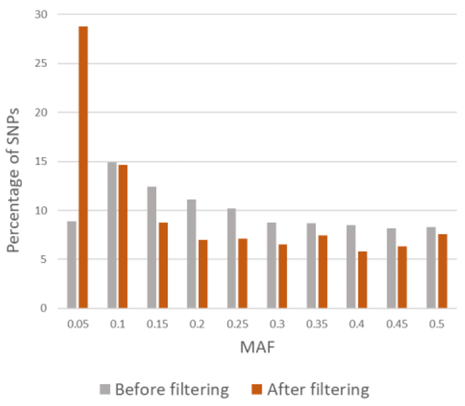

Supplementary Figure S2: Sequence diversity of the barley phenology core collection. a) SNP frequency per 100 Mb across all chromosomes. b) SNP marker efficiency in the panel. Percentage of distribution of MAF of SNPs in the panel before and after filtering. MAF, minor allele frequency

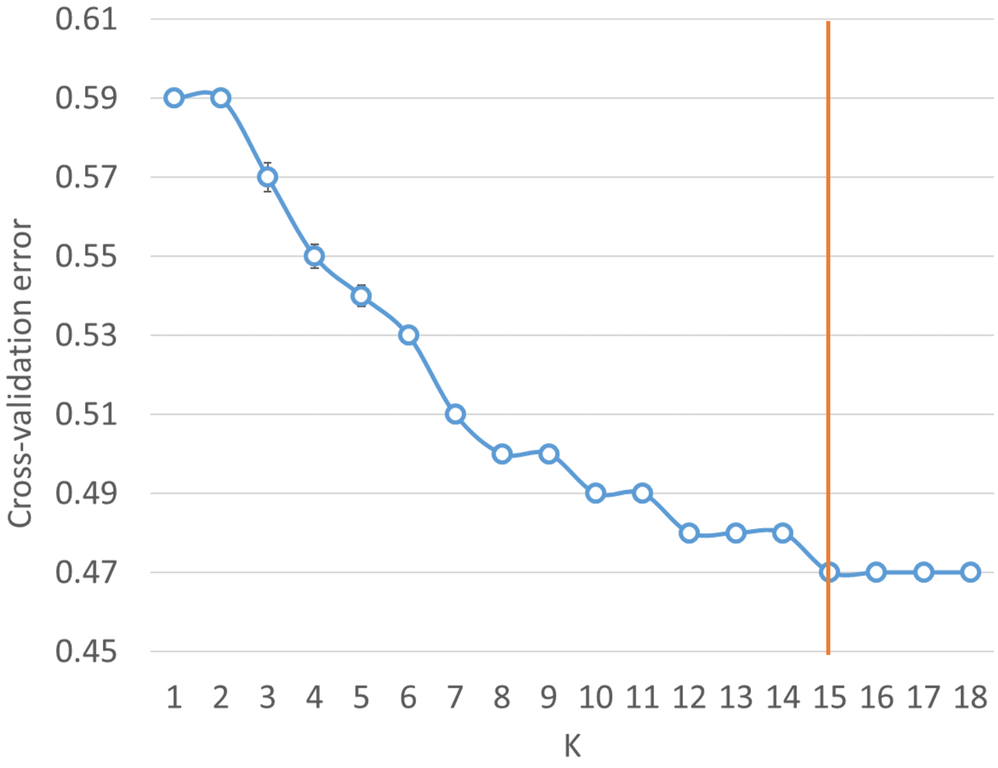

Supplementary Figure S3. Exploration of the optimal number of genetic subpopulations (K) using Δ cross-validation error and standard error values in the barley germplasm collection. A solid line denotes the choice of K=15 which represents the most likely number of subpopulations within the barley germplasm collection.

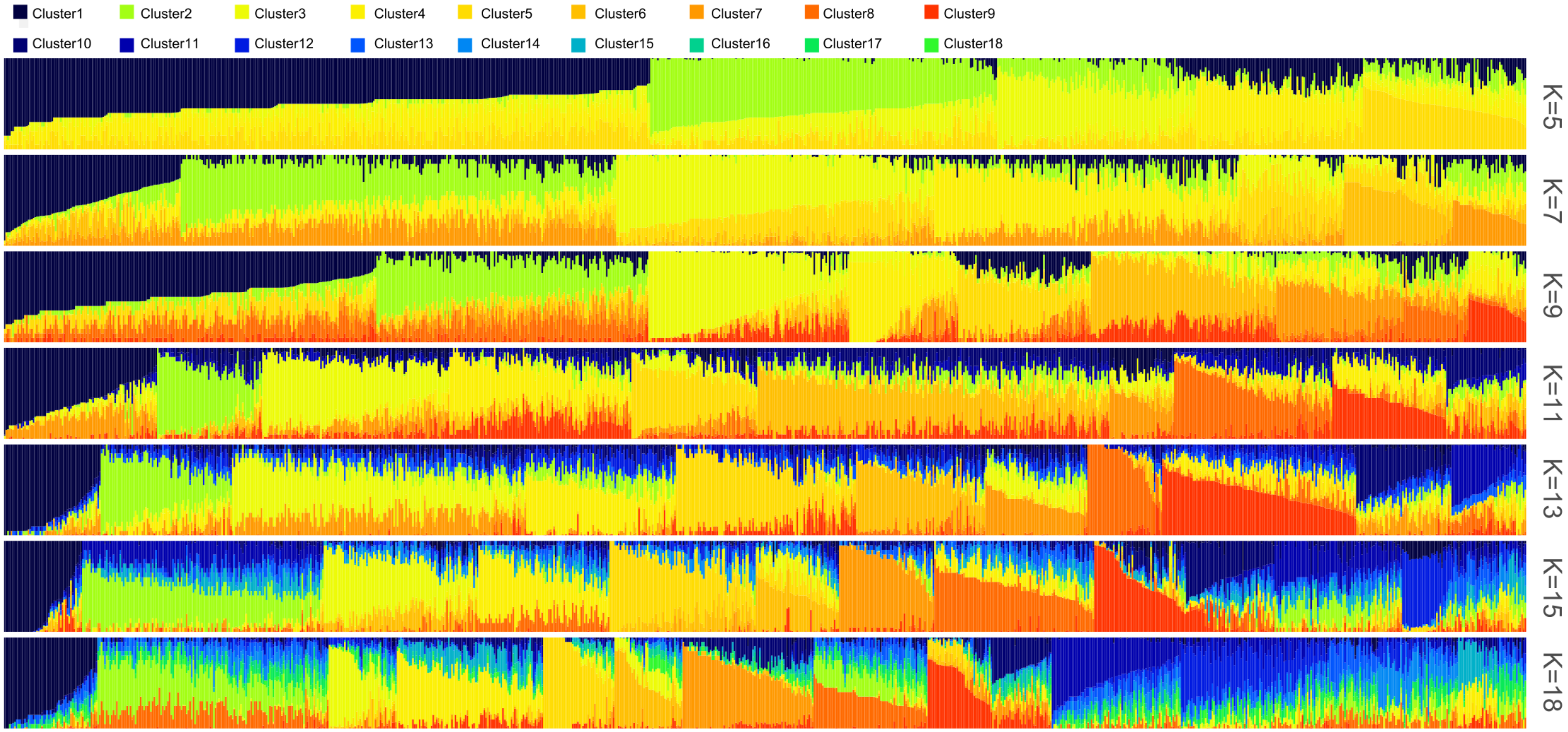

Supplemental Figure S4. Plot of ancestry estimates inferred by ADMIXTURE for 895 worldwide barley accessions for 4,260 SNPs. Each colour represents a population, and the colour of individual haplotypes represents their proportional membership in the different populations. Membership coefficients for each population were merged across 20 replicate runs using the CLUMPP programme. The number of clusters (K) present in the entire population of 895 accessions was judged to be K = 15 based on the CV error. Shown are clusters 5, 7, 9, 11, 13, 15, and 18.

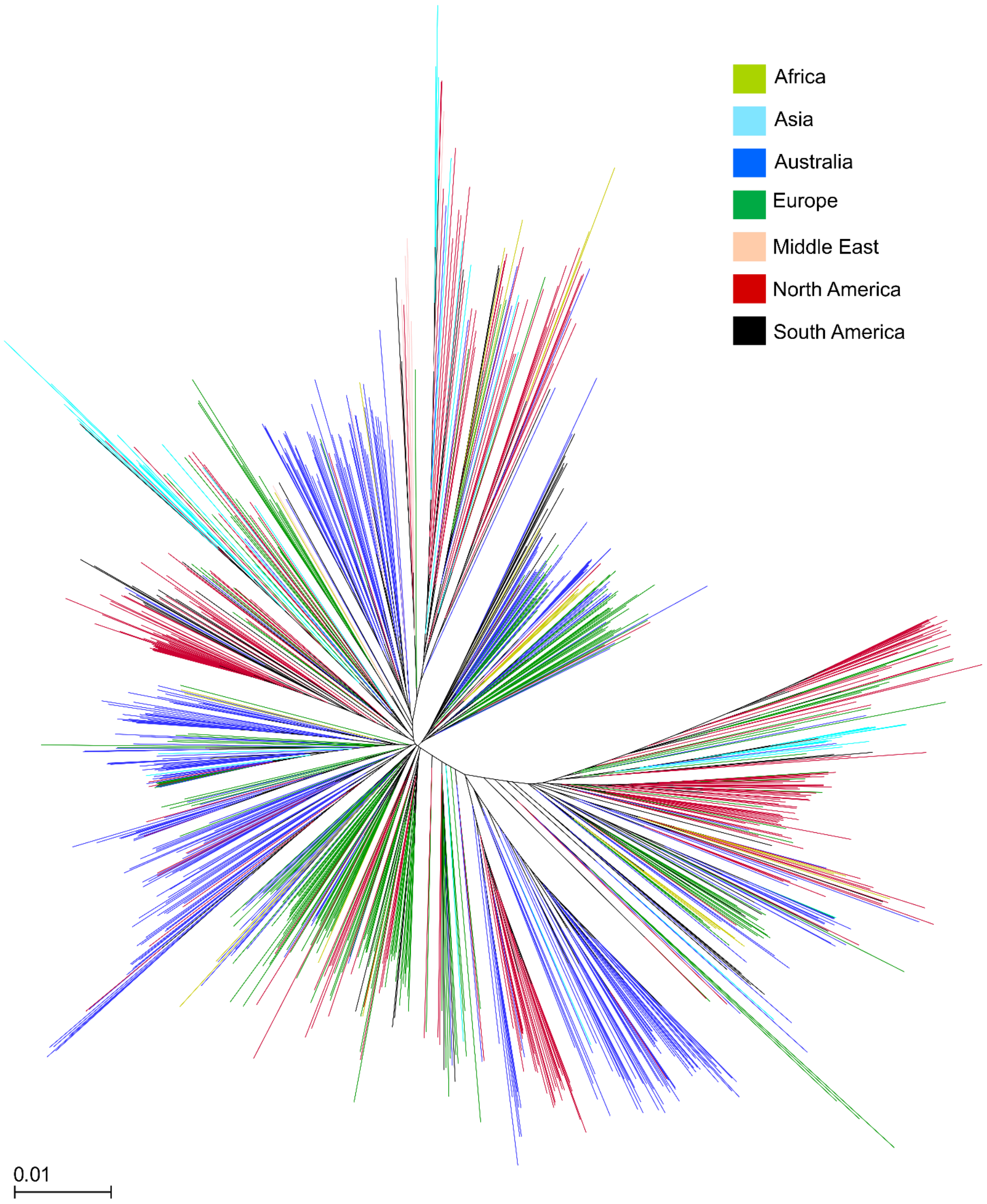

Supplementary Figure S5. Neighbour-joining tree of 895 barley varieties. Each colour represents a population as per legend. The tree was constructed from simple matching distance of 4,260 common SNP in the barley population. Barley variety information is provided in Supplementary Data Set S2.

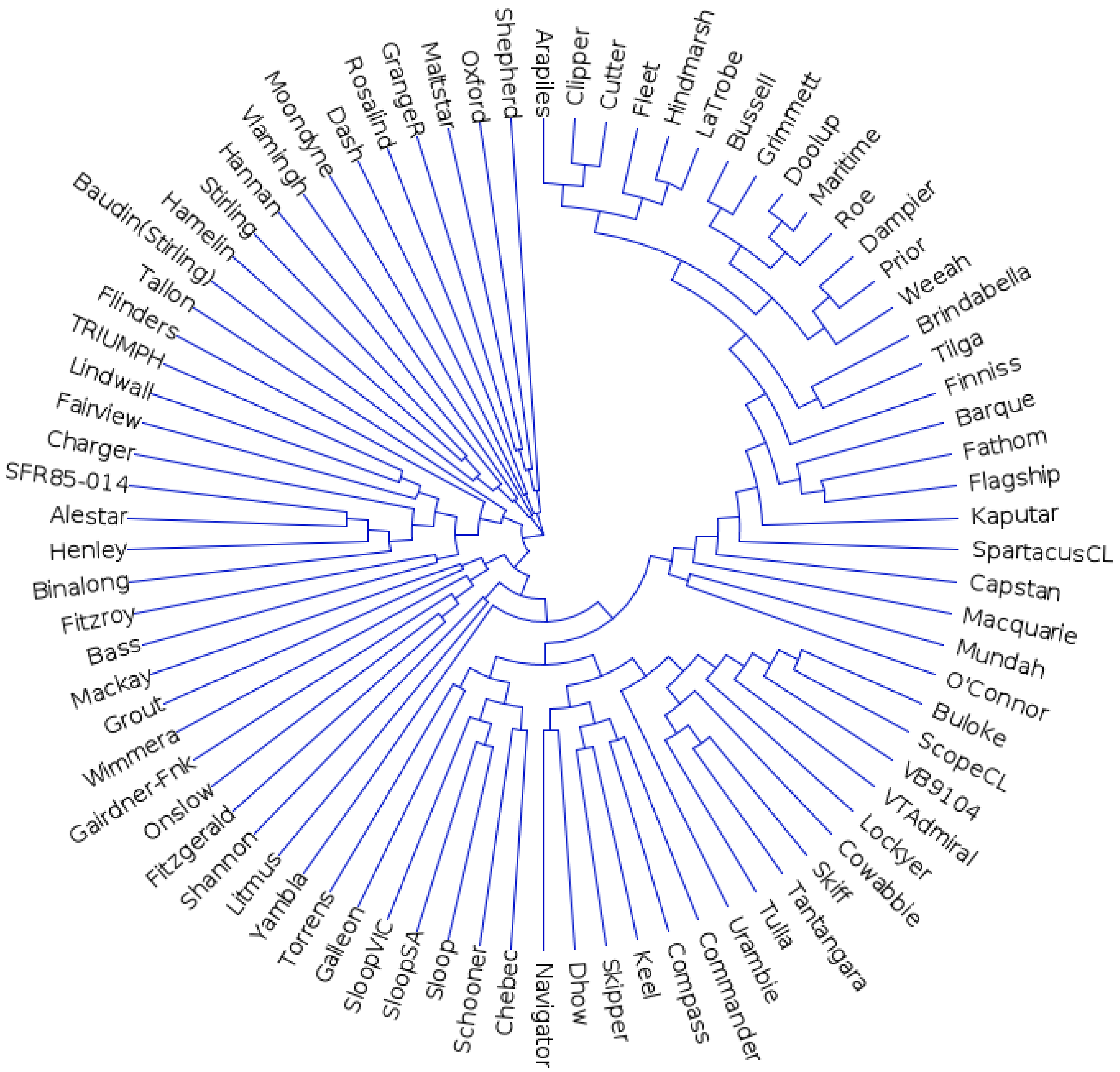

Supplementary Figure S6. Neighbour-joining tree of 82 selected Australian barley varieties. The tree was constructed from simple matching distance of 4,260 common SNP in the barley population. Barley variety information is provided in Supplementary Data Set S2.

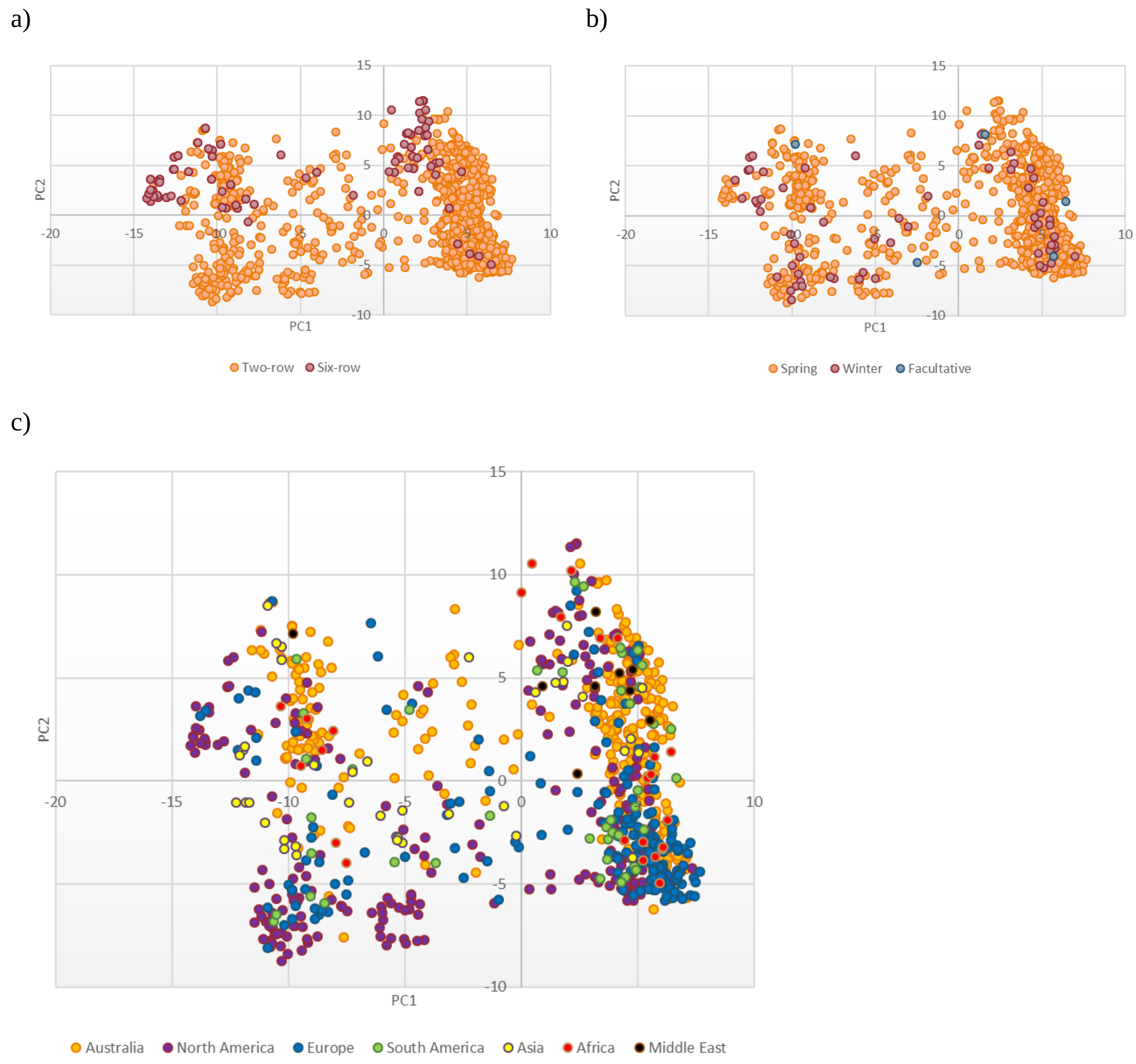

Supplementary Figure S7. **Principal component analysis (PCA) of the first two components of 895 barley varieties.** a) PCA based on row type. The two divergence groups are coloured respectively. b) PCA based on growth habit. The three divergence groups are coloured respectively. c) PCA based on geographic region. The five divergence groups are coloured respectively. PC1 and PC2 together explain about 21.5% of the total variation, and partitioned the population into distinct clusters.

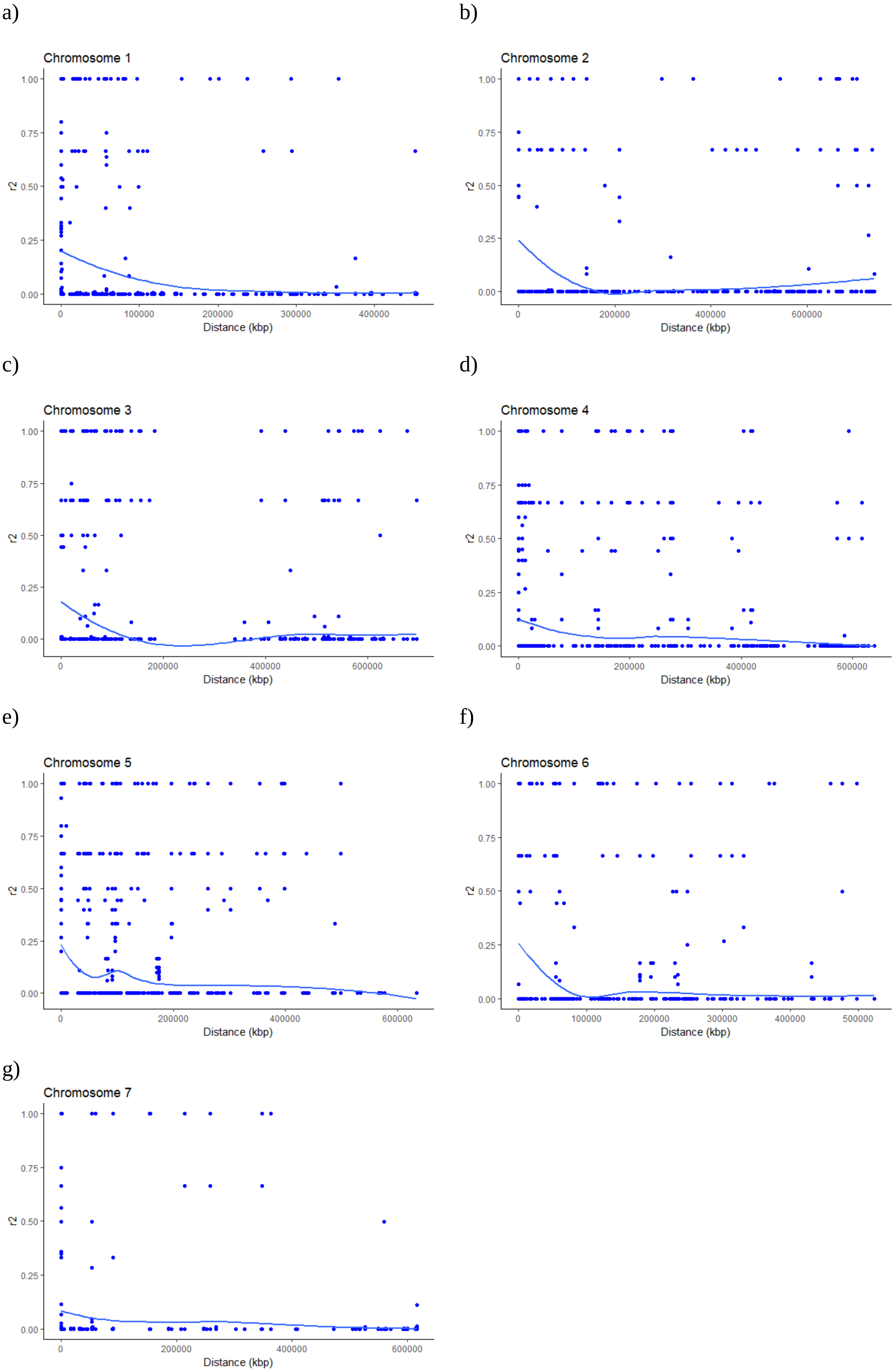
a)
Chromosome 1
r2
1.00
0.75
0.50
0.25
0.00
0
100000
200000
300000
400000
Distance (kbp)
b)
Chromosome 2
r2
1.00
0.75
0.50
0.25
0.00
0
200000
400000
600000
Distance (kbp)
c)
Chromosome 3
r2
1.00
0.75
0.50
0.25
0.00
0
200000
400000
600000
Distance (kbp)
d)
Chromosome 4
r2
1.00
0.75
0.50
0.25
0.00
0
200000
400000
600000
Distance (kbp)
e)
Chromosome 5
r2
1.00
0.75
0.50
0.25
0.00
0
200000
400000
600000
Distance (kbp)
f)
Chromosome 6
r2
1.00
0.75
0.50
0.25
0.00
0
100000
200000
300000
400000
500000
Distance (kbp)
g)
Chromosome 7
r2
1.00
0.75
0.50
0.25
0.00
0
200000
400000
600000
Distance (kbp)

Supplemental Figure S8: The extent of LD in the barley phenology core set of a worldwide collection of domesticated barley varieties. a)-g): Chromosomes 1-7. Values are mean intra-chromosomal LD r^2 values for all intra-chromosomal pairs of SNPs binned by distance. Curves were fit by second-degree loess.

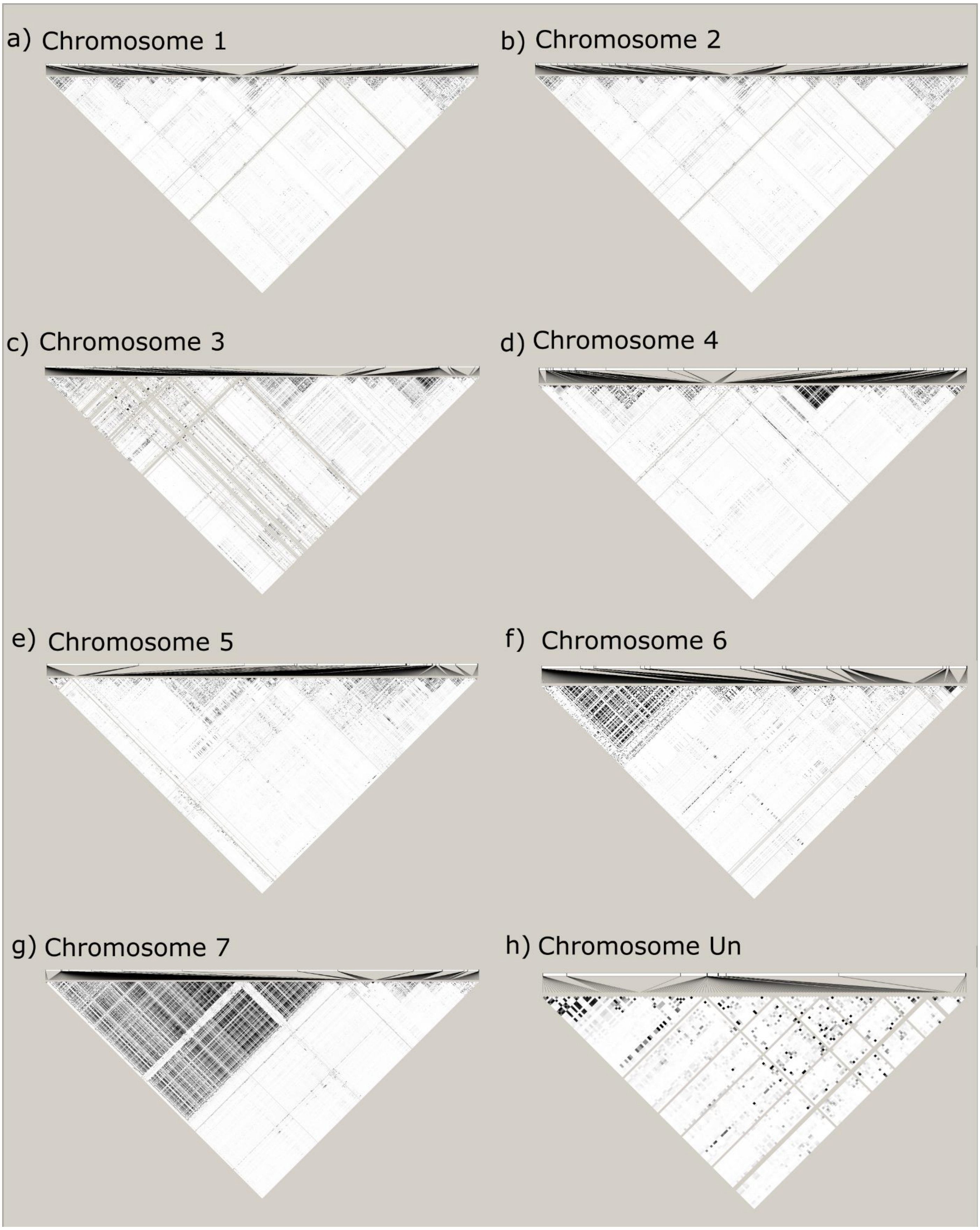

Supplemental Figure S9. **Linkage disequilibrium plots of 4,260 SNPs for 895 barley accessions.** a)-g): Chromosomes 1-7; h) Chromosome Un. LD plots, generated in HAPLOVIEW (Barret et al., 2005), indicate r^2 values between pairs of SNPs multiplied by 100; white, $r^2 = 0$; shades of grey, $0 < r^2 < 1$; black, $r^2 = 1$.

a) Simple model for flowering time

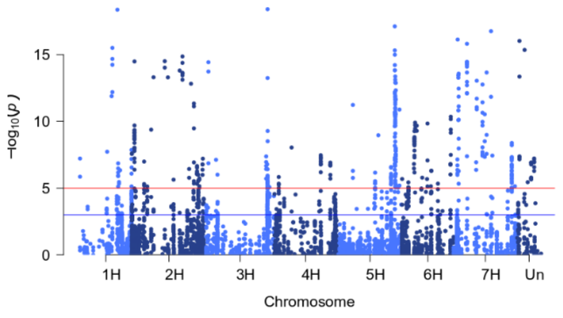

b) Quantile-quantile plot of the simple model

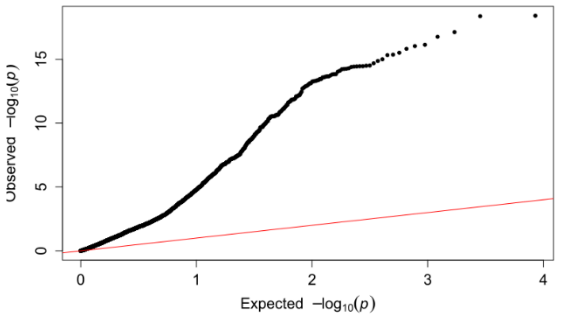

c) MLM model for flowering time

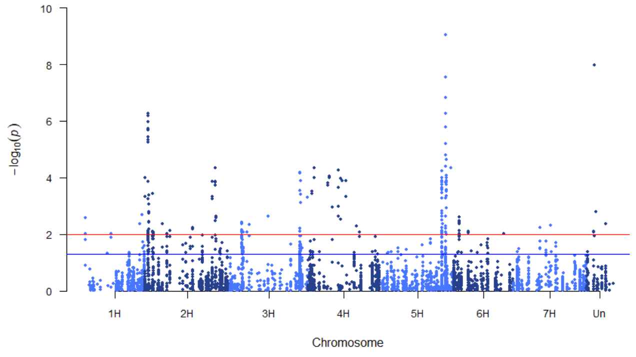

d) Quantile-quantile plot of the MLM model

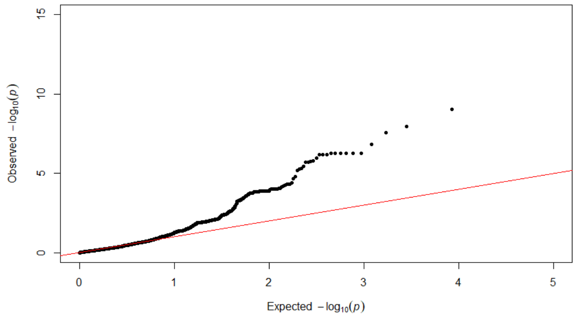

Supplemental Figure S10. Manhattan plots of flowering time for the Esperance 2015 environment. a) Manhattan plot of the naïve model. GWAS results are presented by negative $\log_{10}$ of unadjusted p-values against position on each of the seven chromosomes. Horizontal dashed lines indicate the genome-wide significant threshold set at $1e^{-10-5}$ (blue) and $1e^{-10-3}$ (red). b) Quantile–quantile plot for flowering time based on negative $\log_{10}$ of unadjusted p-values from the naïve model. c) Manhattan plot of the MLM model. GWAS results are presented by negative $\log_{10}$ of FDR adjusted p-values (q-values) against position on each of the seven chromosomes. Horizontal dashed lines indicate the genome-wide significant threshold selected by local false discovery rate and a q-value cut-off at 0.05 (blue) and 0.01 (red). d) Quantile–quantile plot for flowering time based on negative $\log_{10}$ of FDR adjusted p-values (q-value) from the MLM model.

a) Simple model for flowering time

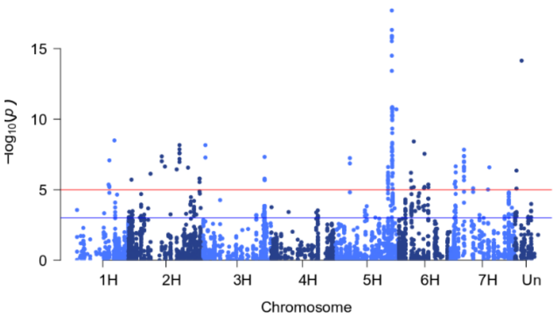

b) Quantile-quantile plot of the simple model

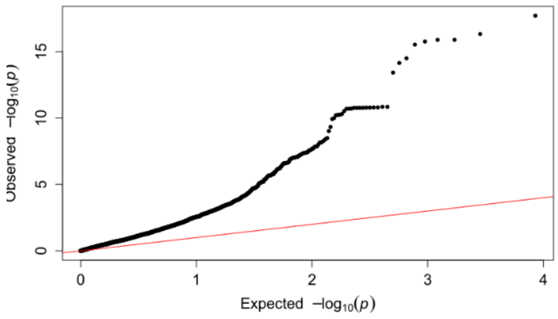

c) MLM model for flowering time

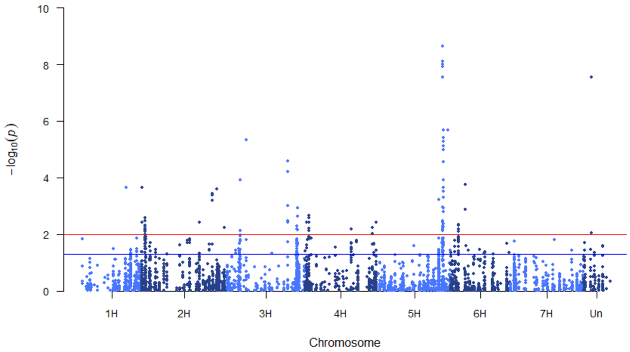

d) Quantile-quantile plot of the MLM model

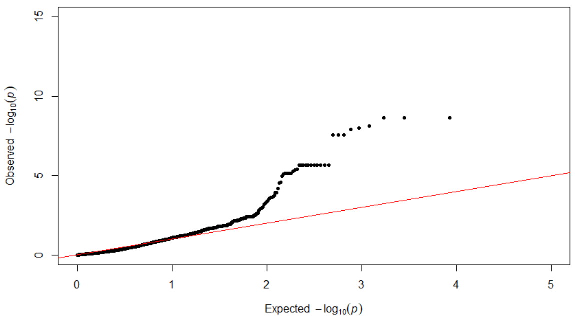

Supplemental Figure S11. Manhattan plots of flowering time for the Esperance 2016 environment.

Other details as per legend to Supplemental Figure 5.

a) Simple model for flowering time

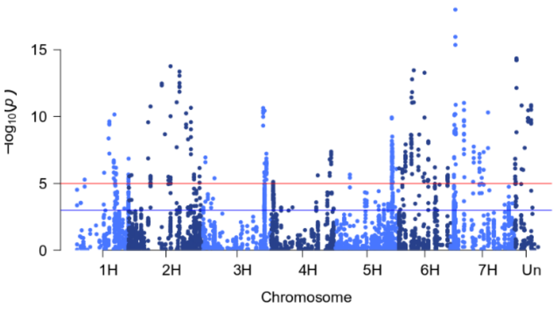

b) Quantile-quantile plot of the simple model

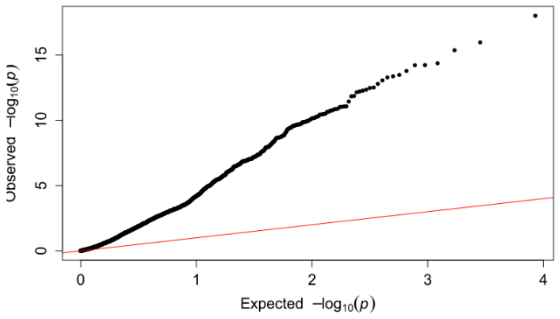

c) MLM model for flowering time

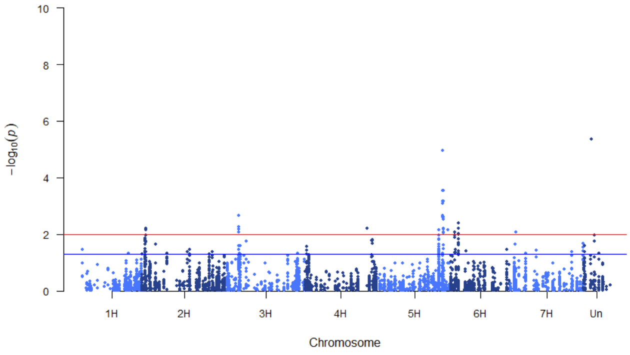

d) Quantile-quantile plot of the MLM model

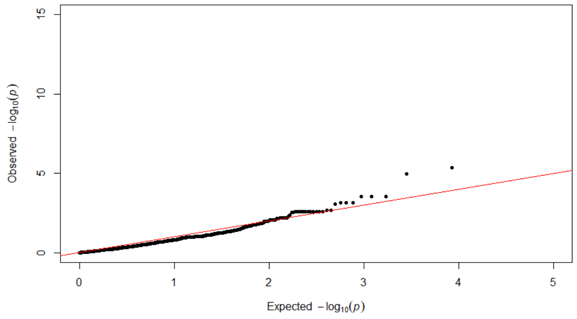

Supplemental Figure S12. Manhattan plots of flowering time for the Geraldton 2015 environment.

Other details as per legend to Supplemental Figure 5.

a) Simple model for flowering time

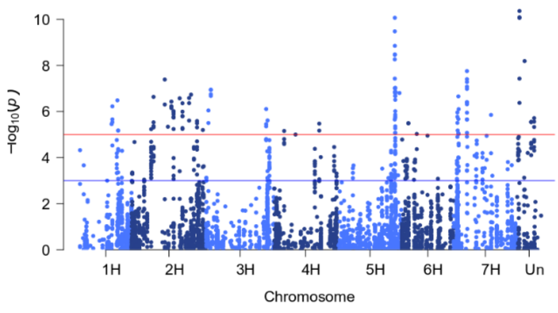

b) Quantile-quantile plot of the simple model

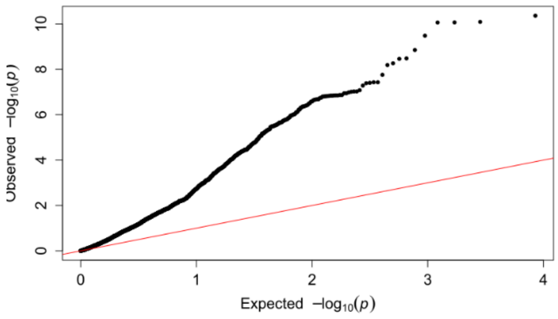

c) MLM model for flowering time

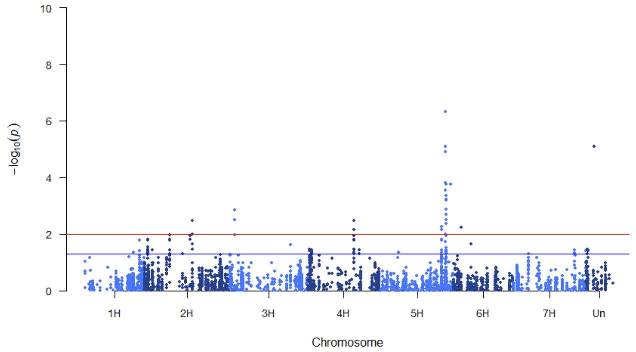

d) Quantile-quantile plot of the MLM model

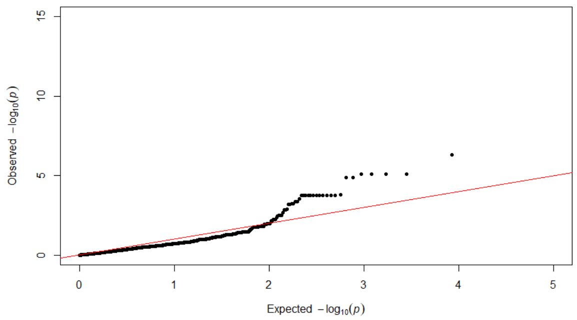

Supplemental Figure S13. Manhattan plots of flowering time for the Geraldton 2016 environment. Other details as per legend to Supplemental Figure 5.

a) Simple model for flowering time

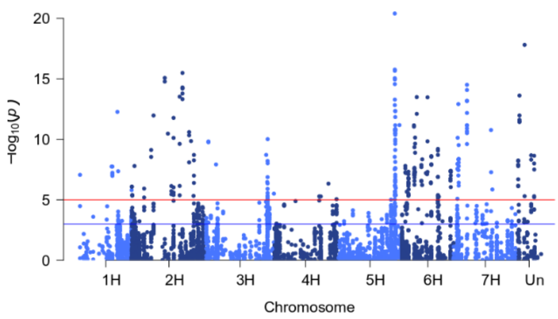

b) Quantile-quantile plot of the simple model

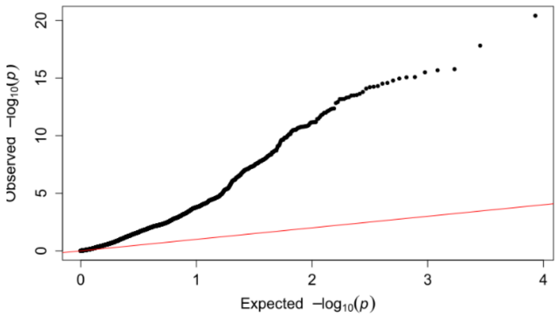

c) MLM model for flowering time

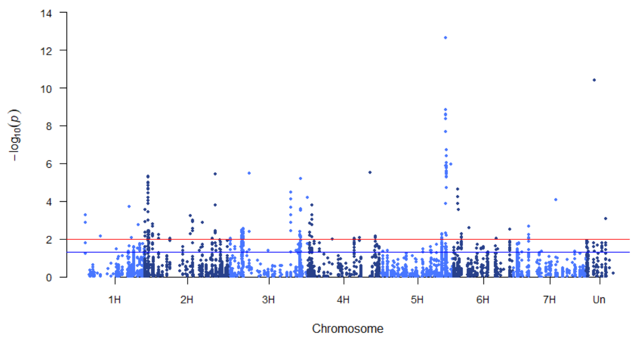

d) Quantile-quantile plot of the MLM model

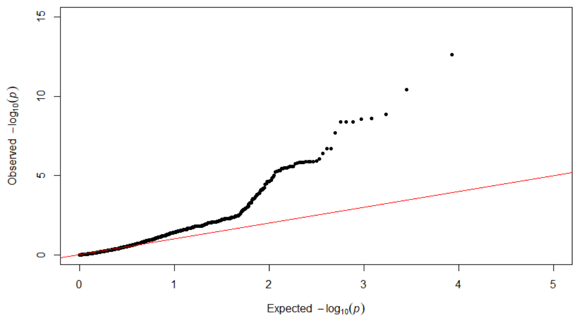

Supplemental Figure S14. Manhattan plots of flowering time for the Katanning 2015 environment. Other details as per legend to Supplemental Figure 5.

a) Simple model for flowering time

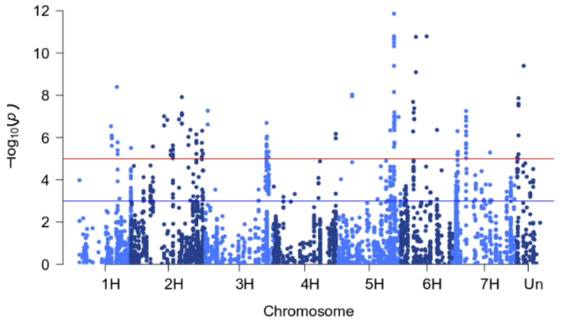

b) Quantile-quantile plot of the simple model

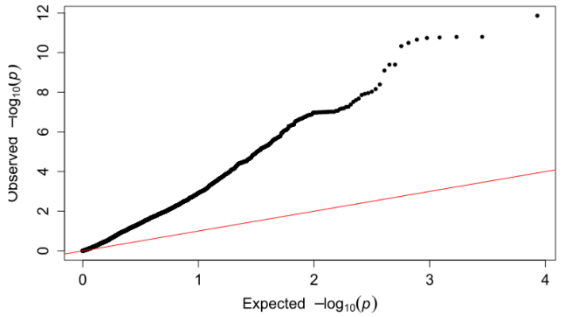

c) MLM model for flowering time

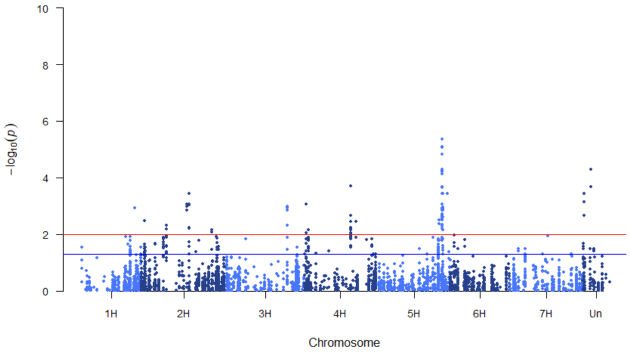

d) Quantile-quantile plot of the MLM model

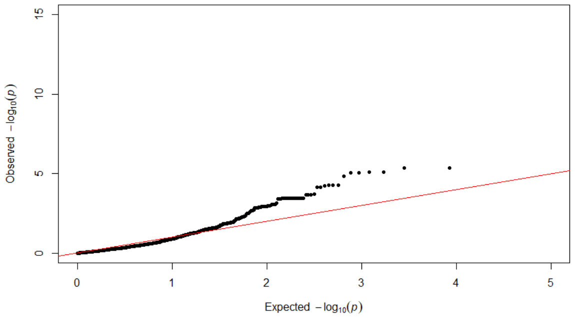

Supplemental Figure S15. Manhattan plots of flowering time for the Katanning 2016 environment. Other details as per legend to Supplemental Figure 5.

a) Simple model for flowering time

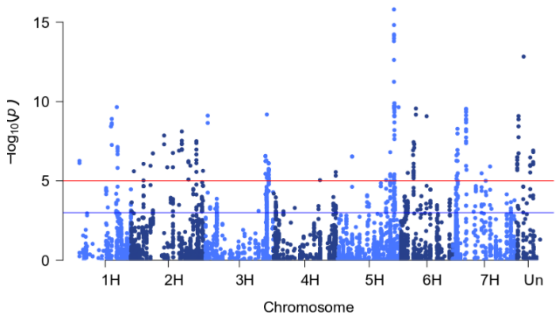

b) Quantile-quantile plot of the simple model

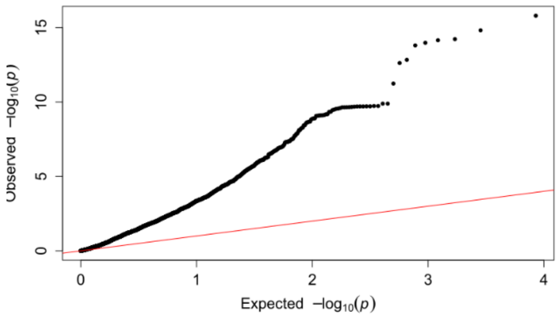

c) MLM model for flowering time

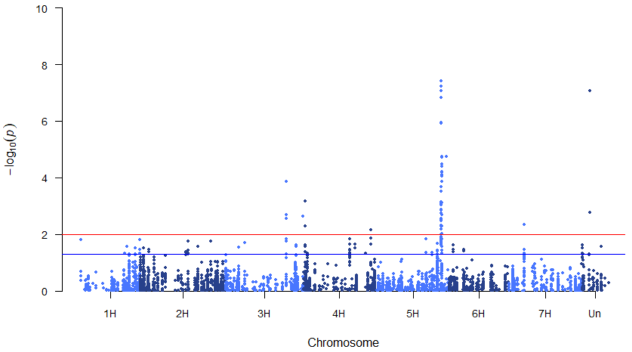

d) Quantile-quantile plot of the MLM model

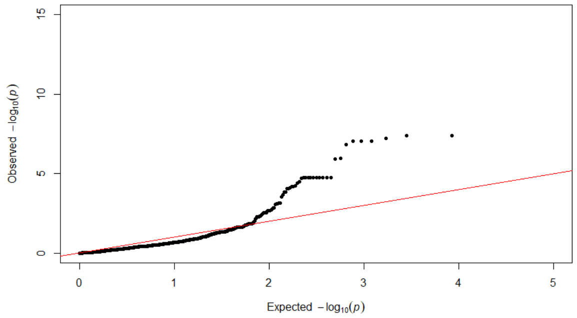

Supplemental Figure S16. Manhattan plots of flowering time for the Merredin 2016 (non-irrigated) environment. Other details as per legend to Supplemental Figure 5.

a) Simple model for flowering time

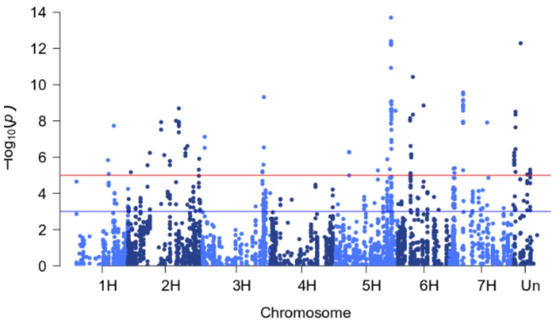

b) Quantile-quantile plot of the simple model

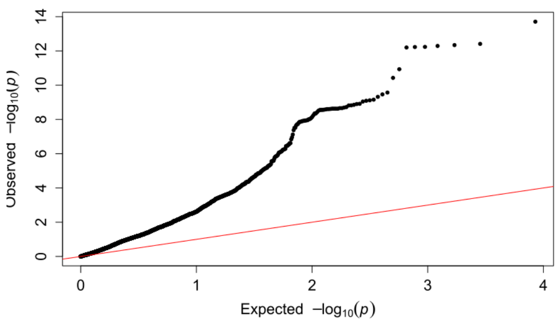

c) MLM model for flowering time

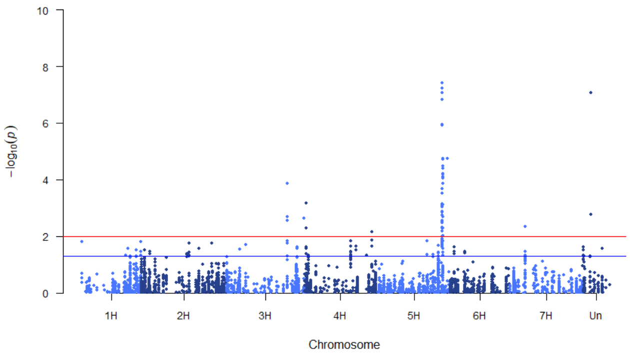

d) Quantile-quantile plot of the MLM model

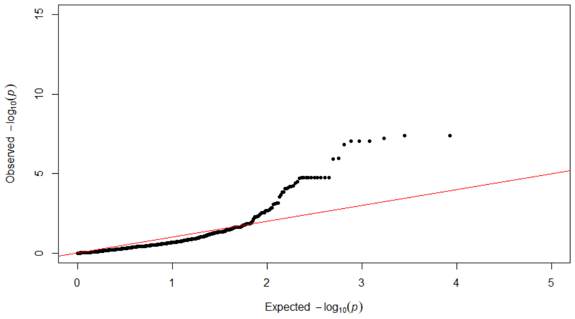

Supplemental Figure S17. Manhattan plots of flowering time for the Merredin 2016 (irrigated) environment. Other details as per legend to Supplemental Figure 5.

a) Simple model for flowering time

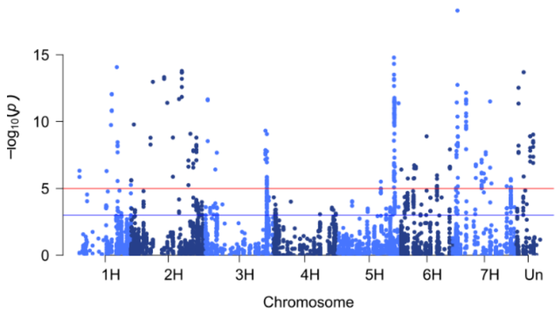

b) Quantile-quantile plot of the simple model

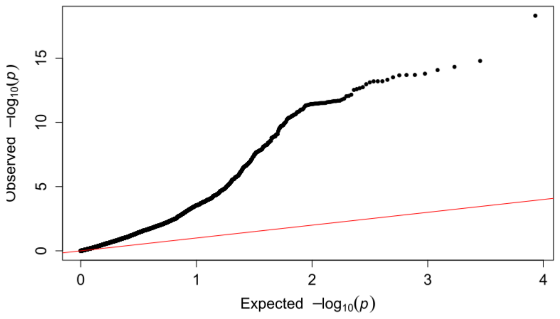

c) MLM model for flowering time

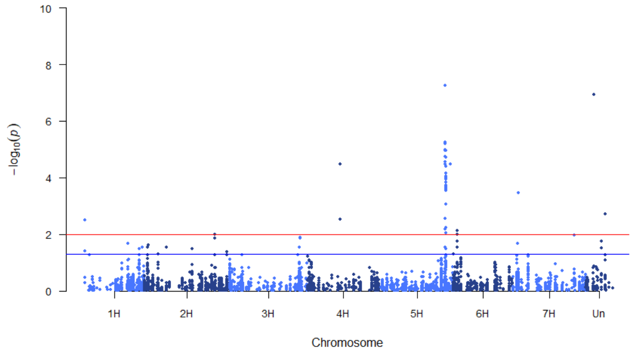

d) Quantile-quantile plot of the MLM model

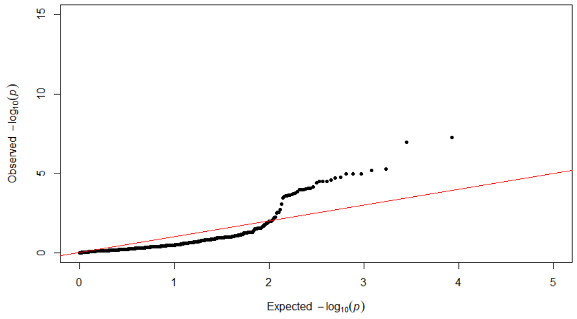

Supplemental Figure S18. Manhattan plots of flowering time for the Perth 2015 (time of planting 1) environment. Other details as per legend to Supplemental Figure 5.

a) Simple model for flowering time

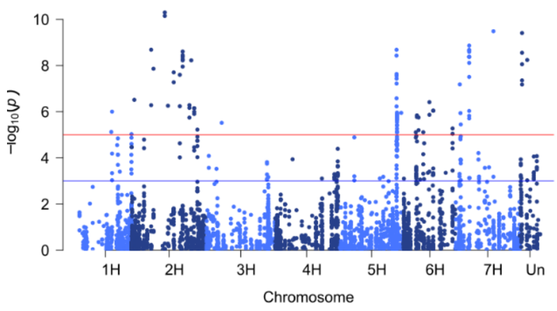

b) Quantile-quantile plot of the simple model

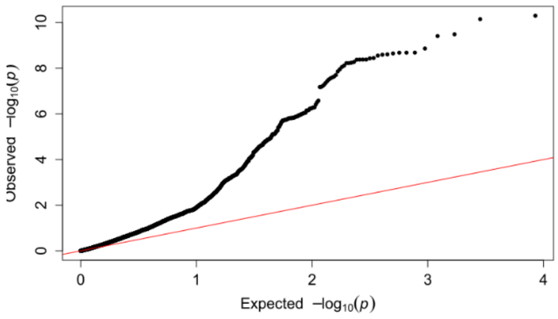

c) MLM model for flowering time

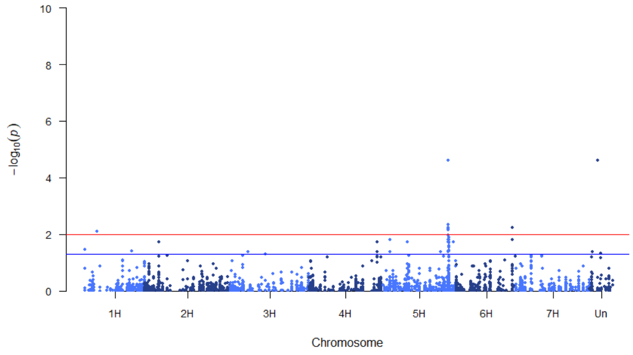

d) Quantile-quantile plot of the MLM model

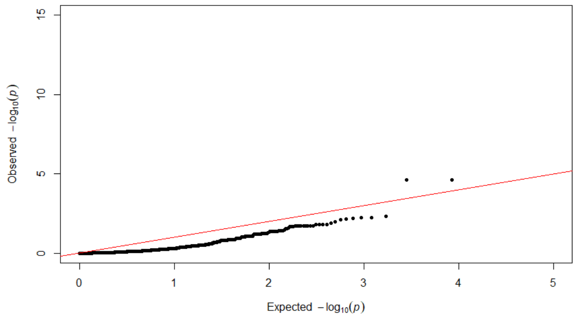

Supplemental Figure S19. Manhattan plots of flowering time for the Perth 2015 (time of planting 2) environment. Other details as per legend to Supplemental Figure 5.

a) Simple model for flowering time

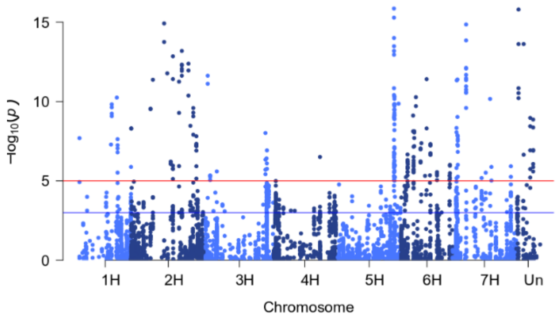

b) Quantile-quantile plot of the simple model

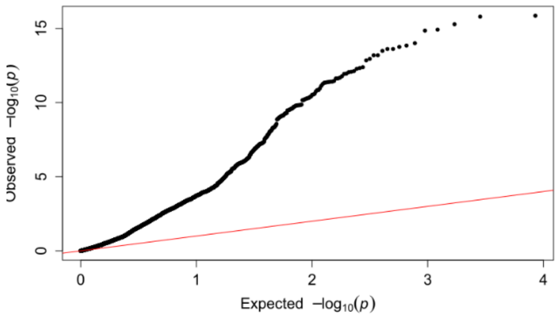

c) MLM model for flowering time

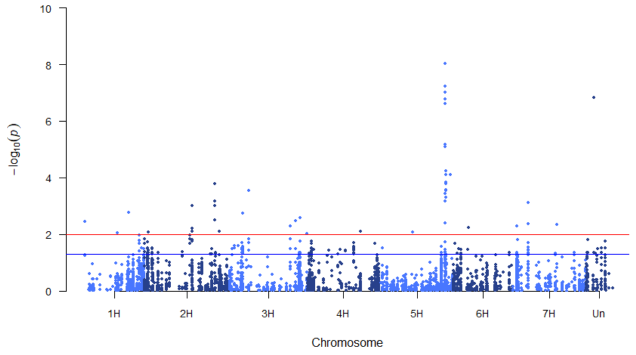

d) Quantile-quantile plot of the MLM model

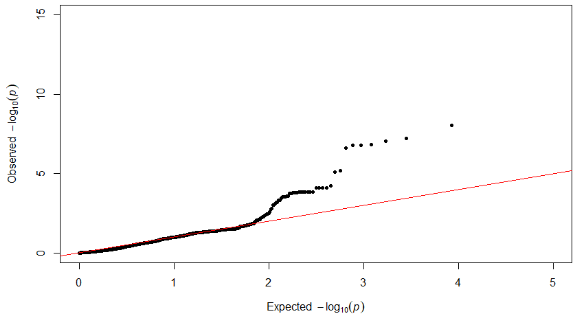

Supplemental Figure S20. Manhattan plots of flowering time for the Perth 2015 (time of planting 2) environment. Other details as per legend to Supplemental Figure 5.

a) Simple model for flowering time

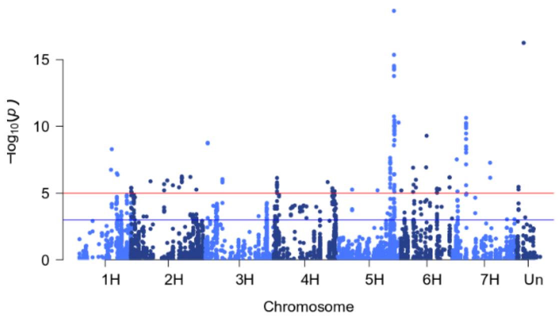

b) Quantile-quantile plot of the simple model

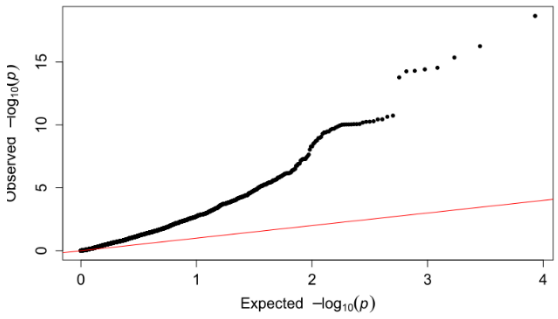

c) MLM model for flowering time

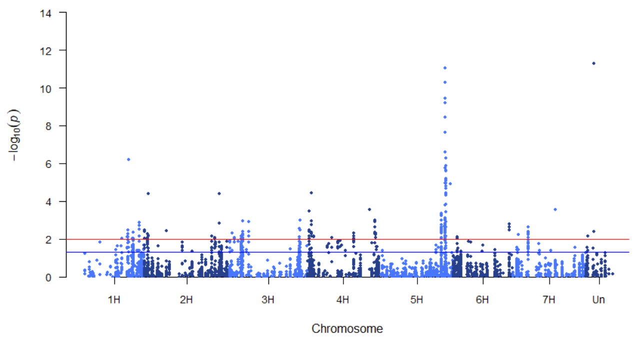

d) Quantile-quantile plot of the MLM model

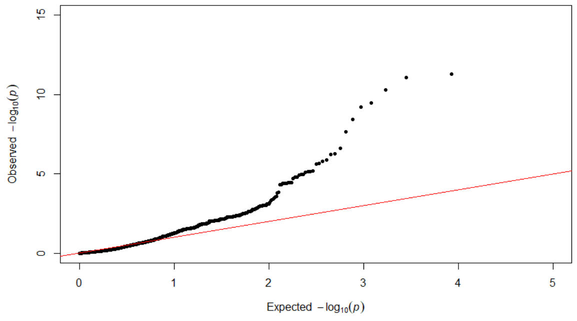

Supplemental Figure S21. Manhattan plots of flowering time for the Perth 2016 environment. Other details as per legend to Supplemental Figure 5.

a) Simple model for grain yield

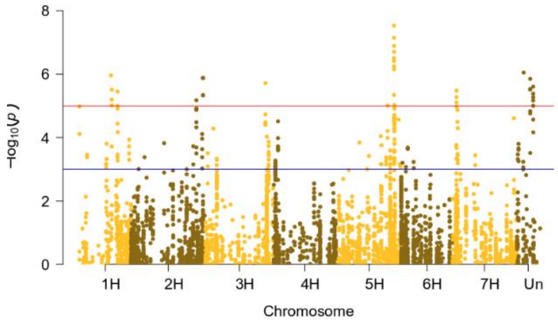

b) Quantile-quantile plot of the simple model

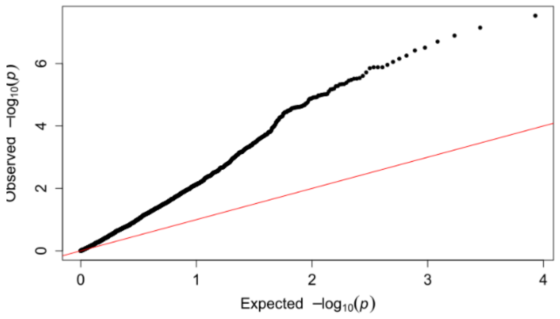

c) MLM model for grain yield

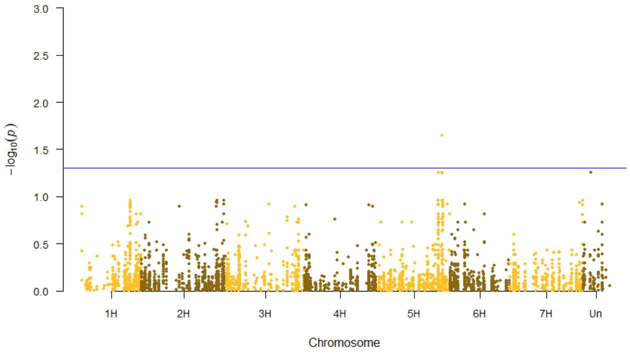

d) Quantile-quantile plot of the MLM model

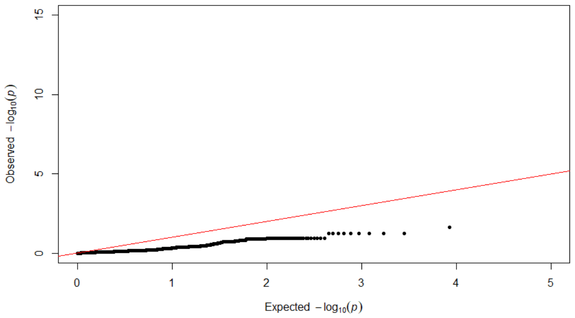

Supplemental Figure S22. Manhattan plots of grain yield for the Esperance 2016 environment.

Other details as per legend to Supplemental Figure 5.

a) Simple model for flowering time

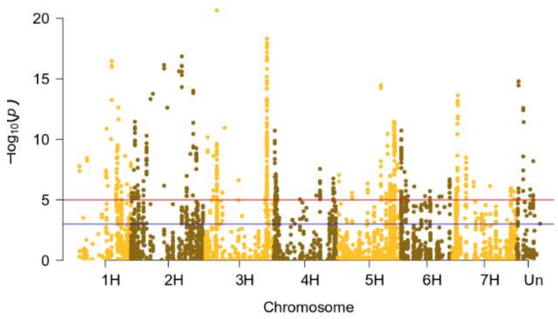

b) Quantile-quantile plot of the simple model

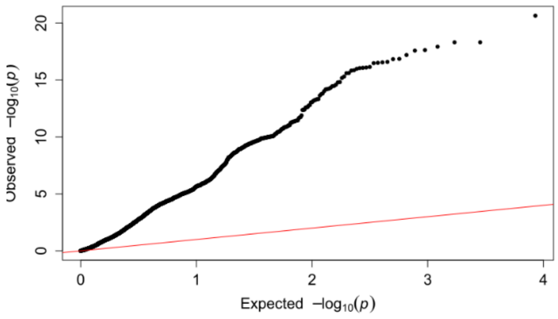

c) MLM model for flowering time

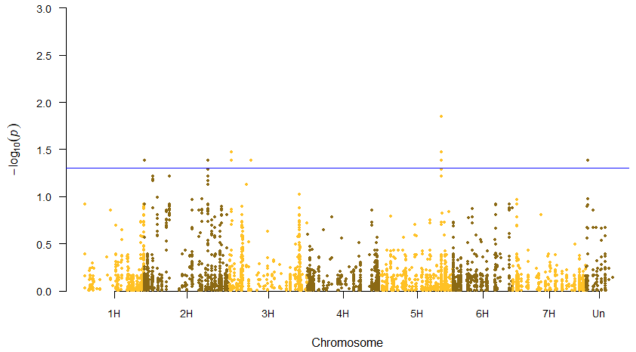

d) Quantile-quantile plot of the MLM model

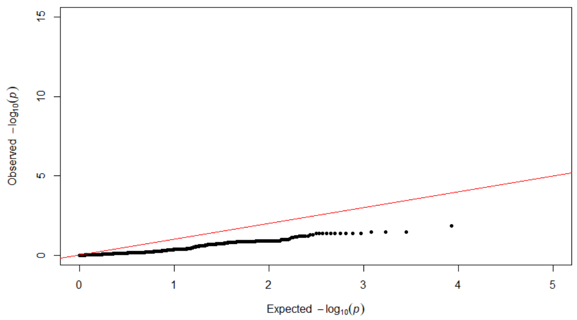

Supplemental Figure S23. Manhattan plots of grain yield for the Katanning 2015 environment.

Other details as per legend to Supplemental Figure 5.

a) Simple model for grain yield

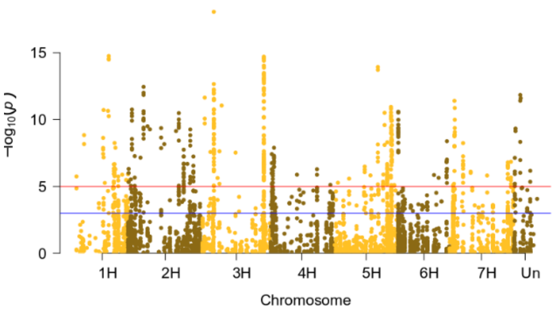

b) Quantile-quantile plot of the simple model

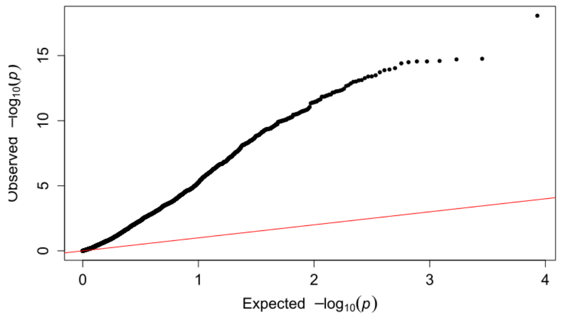

c) MLM model for grain yield

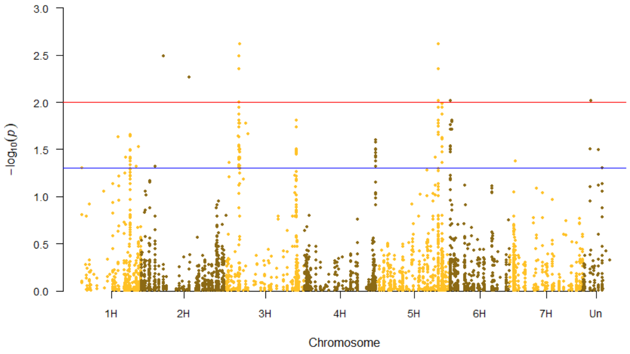

d) Quantile-quantile plot of the MLM model

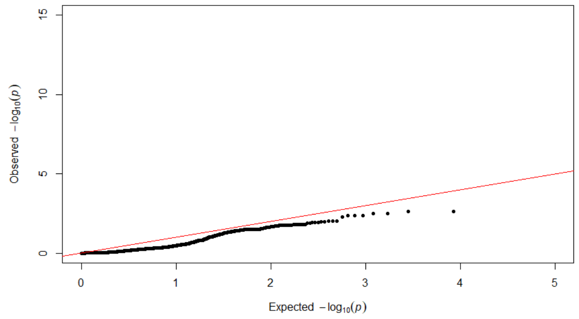

Supplemental Figure S24. Manhattan plots of grain yield for the Merredin 2016 (non-irrigated) environment. Other details as per legend to Supplemental Figure 5.

a) Simple model for grain yield

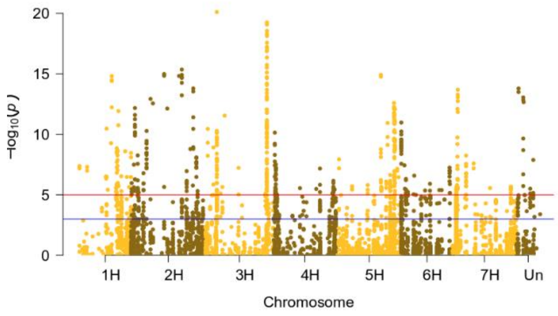

b) Quantile-quantile plot of the simple model

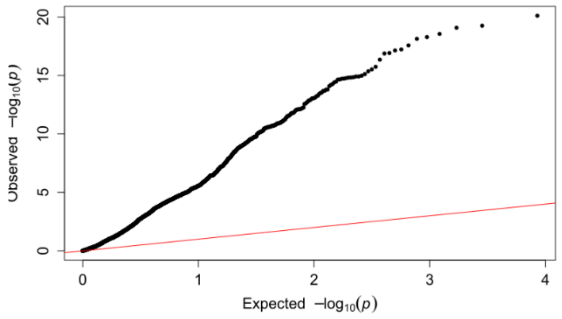

c) MLM model for grain yield

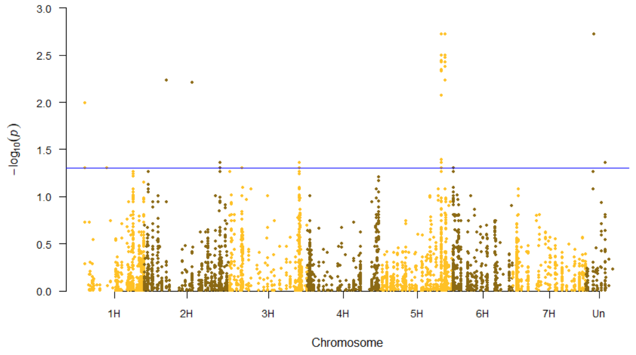

d) Quantile-quantile plot of the MLM model

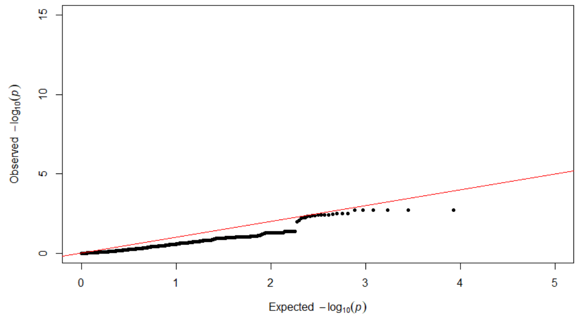

Supplemental Figure S25. Manhattan plots of grain yield for the Merredin 2016 (irrigated) environment. Other details as per legend to Supplemental Figure 5.

a) Simple model for plant height

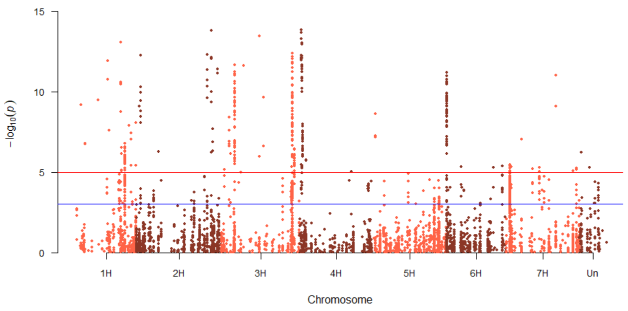

b) Quantile-quantile plot of the simple model

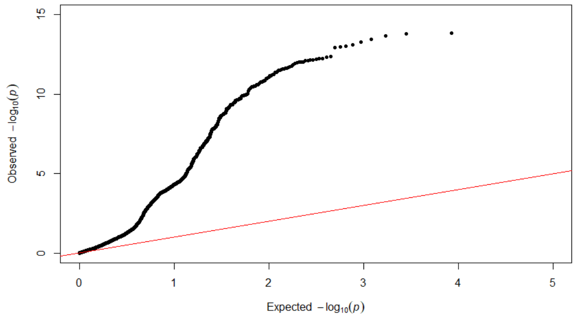

c) MLM model for plant height

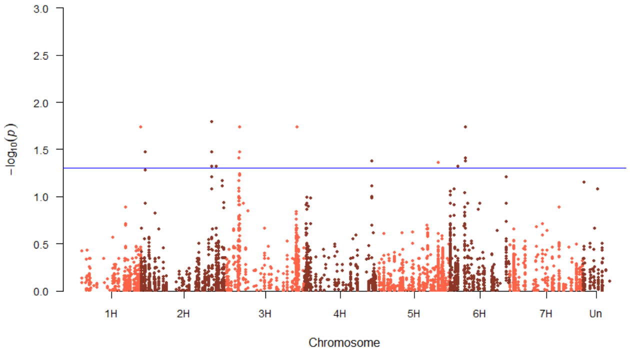

d) Quantile-quantile plot of the MLM model

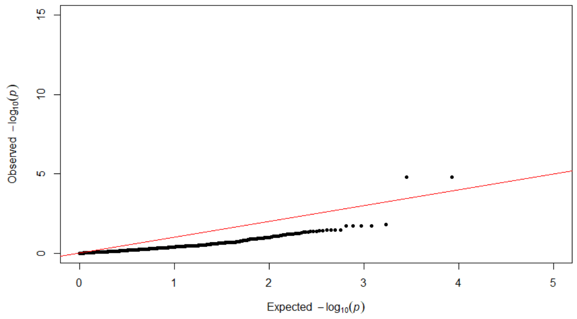

Supplemental Figure S26. Manhattan plots of plant height for the Geraldton 2015 environment.

Other details as per legend to Supplemental Figure 5.

a) Simple model for plant height

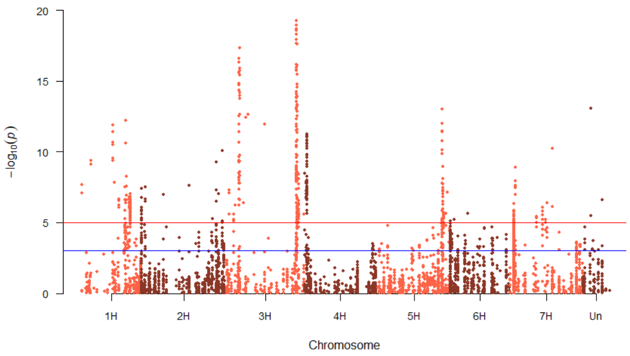

b) Quantile-quantile plot of the simple model

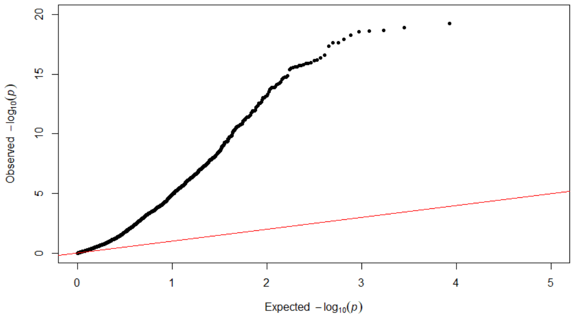

c) MLM model for plant height

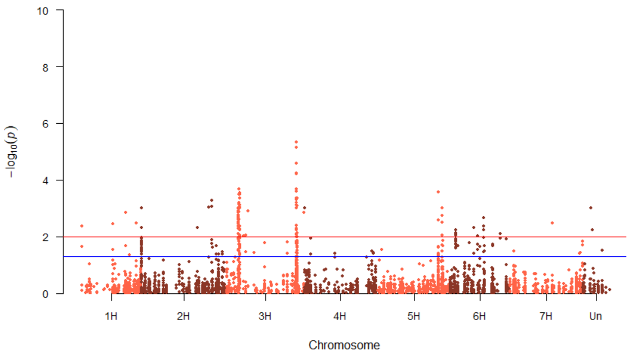

d) Quantile-quantile plot of the MLM model

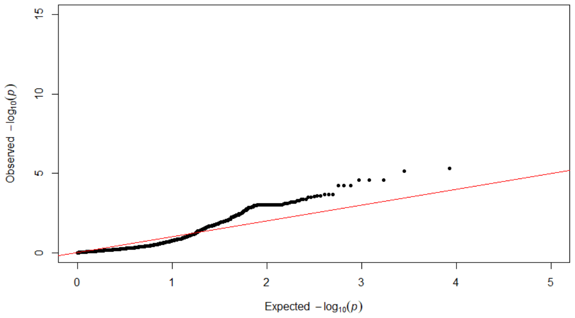

Supplemental Figure S27. Manhattan plots of plant height for the Katanning 2015 environment.

Other details as per legend to Supplemental Figure 5.

a) Simple model for plant height

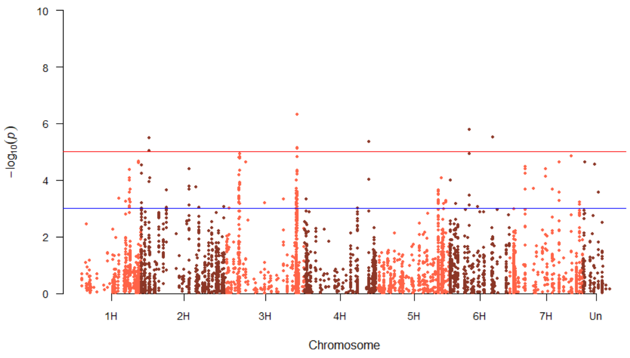

b) Quantile-quantile plot of the simple model

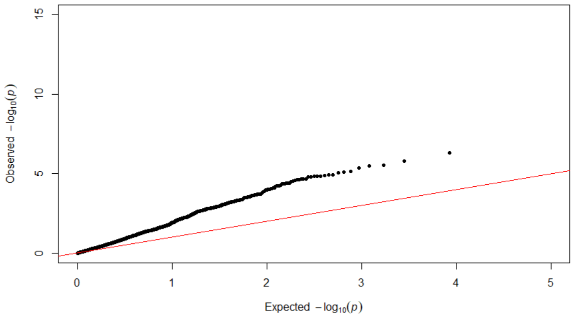

c) MLM model for plant height

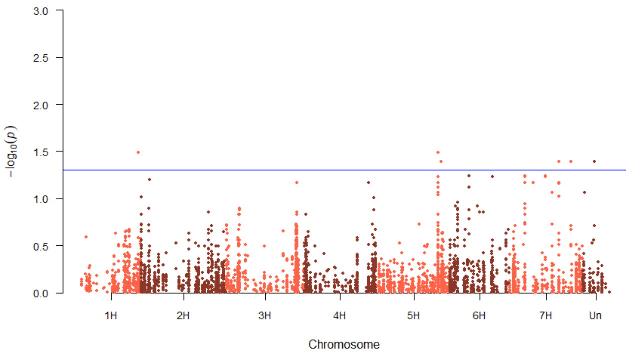

d) Quantile-quantile plot of the MLM model

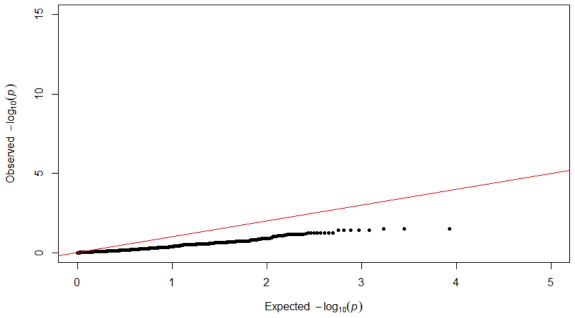

Supplemental Figure S28. Manhattan plots of plant height for the Perth 2016 environment. Other details as per legend to Supplemental Figure 5.

a) Simple model for plant height

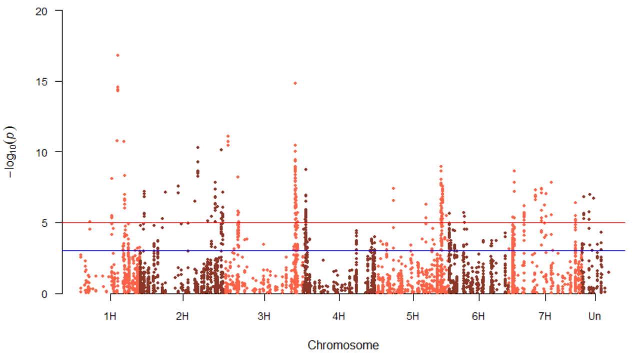

b) Quantile-quantile plot of the simple model

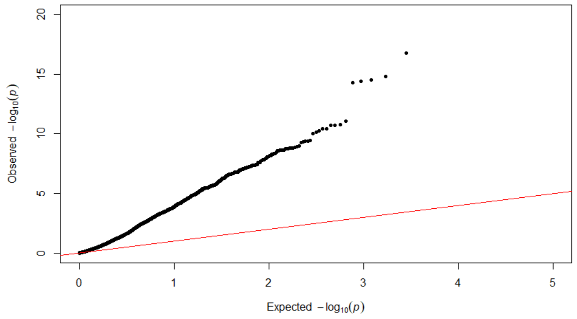

c) MLM model for plant height

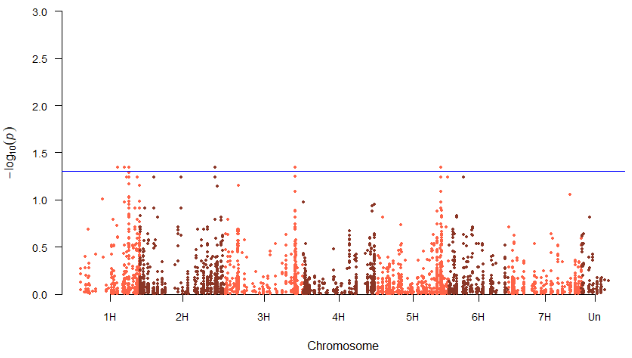

d) Quantile-quantile plot of the MLM model

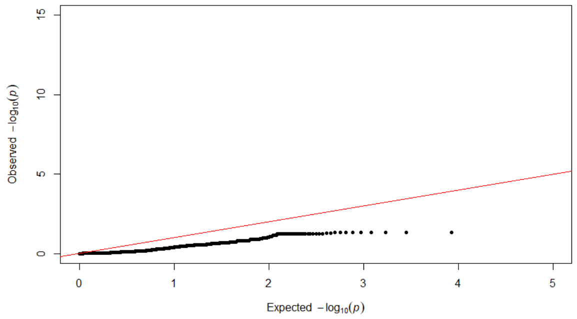

Supplemental Figure S29. Manhattan plots of plant height for the Merredin 2016 (non-irrigated) environment. Other details as per legend to Supplemental Figure 5.

a) Simple model for plant height

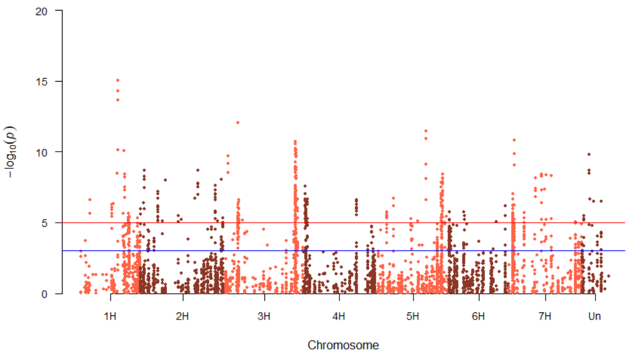

b) Quantile-quantile plot of the simple model

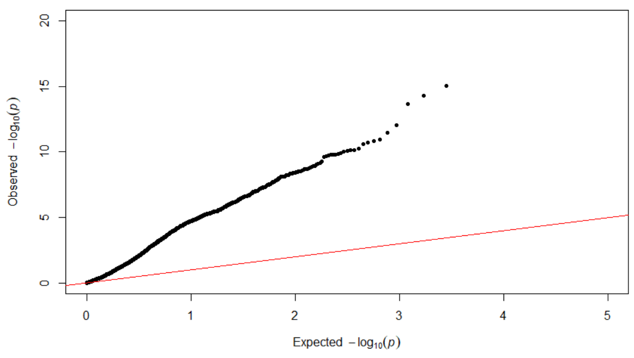

c) MLM model for plant height

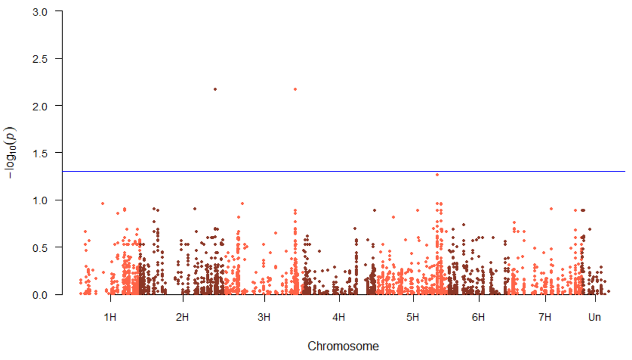

d) Quantile-quantile plot of the MLM model

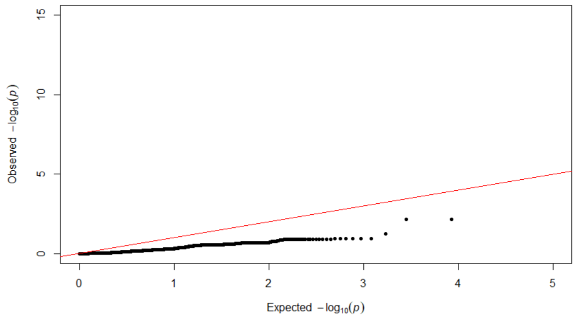

Supplemental Figure S30. Manhattan plots of plant height for the Merredin 2016 (irrigated) environment. Other details as per legend to Supplemental Figure 5.
