# Supplementary material for: Hybridisation‐based target enrichment of phenology genes to dissect the genetic basis of yield and adaptation in barley
Source: Plant Biotechnol J. 2018 Dec 6;17(5):932–44. doi: 10.1111/pbi.13029 (PMC6587706; doi:10.1111/pbi.13029)
Supplement: Supplementary file 2 — Table S1. The extent of LD in the barley phenology core panel. Table S2. Field site location and climate data during growth period. Table S3. Estimation of mean, minimum (min), maximum (max), median (med), and heritability (h 2) of all traits. Table S4. Functional allele diversity between Chevallier and Prior based on significant SNP‐associations with phenology. Table S5. Functional allele diversity between Prior and Clipper based on significant SNP‐associations with phenology. Table S6. Functional allele diversity between Clipper and La Trobe based on significant SNP‐associations with phenology. Table S7. Functional allele diversity between Chevallier and Prior based on significant SNP‐associations with grain yield. Table S8. Functional allele diversity between Prior and Clipper based on significant SNP‐associations with grain yield. Table S9. Functional allele diversity between Prior and Clipper based on significant SNP‐associations with plant height. Table S10. Functional allele diversity between Clipper and La Trobe based on significant SNP‐associations with plant height. [file PBI-17-932-s008.pdf]

Supplemental Table S1: The extent of LD in the barley phenology core panel. Values are mean LD r^2 values for pairs calculated per chromosome of SNPs binned by distance.

**Distance (Mb)**	**Total pairs**	**Mean r^2**	**Pairs in complete LD**	**% of pairs in LD >0.8**	**% of pairs in LD >0.2**	**Mean of r^2>0.2**
Total	36,727	0.068	1,754	4.78	7.40	0.871
0-10	3,641	0.29	787	21.64	33.48	0.86
10-20	54	0.37	14	25.9	42.6	0.88
20-30	1	0.67	0	0	100	0.67
30-40	5	0	0	0	0	0
40-50	0	0	0	0	0	0
0-50	10,949	0.146	1,175	10.74	17.07	0.85
50-100	4,175	0.080	226	5.41	9.8	0.81
100-150	3,316	0.049	103	3.1	5.82	0.82
150-200	2,279	0.033	38	1.67	2.94	0.82
200-250	1,628	0.025	30	1.84	2.95	0.84
250-300	1,840	0.027	26	1.4	3.48	0.77
300-350	1,280	0.012	9	0.7	1.48	0.76
350-400	1,701	0.023	25	1.47	2.7	0.83
400-450	1,757	0.026	29	1.65	2.96	0.85
450-500	1,303	0.012	6	0.46	1.46	0.75
500-550	2,758	0.019	44	1.56	1.96	0.94
550-600	1,887	0.008	10	0.53	0.95	0.81
600-650	1,283	0.013	14	1.09	1.48	0.9
650-700	394	0.068	18	4.57	8.38	0.81
700-750	176	0.025	0	0.57	3.98	0.49

Supplemental Table S2: Field site location and climate data during growth period. TOP: time of planting. NI: non-irrigate. IR: irrigated

Field site	Year	Latitude	Longitude	Temp Min (˚C)	Temp Max (˚C)	Temp average (˚C)	Global solar exposure average (MJ m^{-2})	Rainfall total (mm)	Rainfall average (mm)	Growth period (days)	Planting date	Harvest date
Esperance	2015	33°36'32.35"S	121°46'23.81"E	4	41	14.71	12.35	318.2	1.57	203	13/05/2015	2/12/2015
	2016	33°36'42.82"S	121°47'29.72"E	4	37	13.57	12.86	343.6	1.71	201	19/05/2016	06/12/2016
Geraldton	2015	28°46'46.19"S	114°39'26.51"E	2	40	17.33	18.03	189.8	1.04	182	28/05/2015	25/11/2015
	2016	28°41'1.13"S	114°44'44.89"E	3	41	15.14	17.5	355.4	1.69	210	12/05/2016	8/12/2016
Katanning	2015	33°41'22.06"S	117°34'42.94"E	2	36	14	14.63	550	2.64	208	15/05/2015	9/12/2015
	2016	33°41'9.36"S	117°37'49.33"E	1	38	12.9	16.87	256.2	1.05	244	01/06/2016	30/12/2015
Perth*	2015	31°59'25.2"S	115°53'04.2"E	0	39	16.29	15.96	489	2.45	200	01/05/2015	16/11/2015
	2015	31°59'25.2"S	115°53'04.2"E	0	41	17.13	16.7	422.4	2.11	200	22/05/2015	7/12/2015
	2015	31°57'13.4"S	115°47'37.7"E	0	41	17.43	18.49	411.2	2.06	200	11/06/2015	27/12/2015
	2016	31°59'25.2"S	115°53'04.2"E	1	42	15.13	15.9	576.6	2.89	200	18/05/2015	3/12/2016
Merredin	2016	31°30'18.43"S	118°13'27.63"E	1	37	14.14	16.39	181.4	0.95	191	16/05/2016	23/11/2015
	2016	31°30'18.43"S	118°13'27.63"E	1	37	14.14	16.39	181.4	0.95	191	16/05/2016	23/11/2016

*Grain yield was not measured at the Perth location; environmental data was taken over 200 days for comparisons.

Supplemental Table S3: Estimation of mean, minimum (min), maximum (max), median (med), and heritability (h^2) of all traits. Heritability was calculated on entry mean basis. GY: Grain yield (kg ha^{-1}), PH: Plant height (cm), NI: non-irrigated, IR: irrigated

**Field site**	**Year**	**Accessions**	**Days to Z49 (min)**	**Days to Z49 (med)**	**Days to Z49 (max)**	**h^2**	**GY (min)**	**GY (med)**	**GY (max)**	**h^2**	**PH (min)**	**PH (med)**	**PH (max)**	**h^2**
Esperance	2015	805	60	104	136	0.68	504	2,636	6,280	0.64	35	80	125	0.36
	2016	459	72	110	146	0.19	520	2,281	5,235	0.53	40	55	85	0.59
Geraldton	2015	811	46	72	89	0.47	510	2,083	3,699	0.53	30	60	90	0.74
	2016	441	47	80	91	0.74	620	3,105	6,309	0.78	40	80	120	0.52
Katanning	2015	813	69	105	132	0.59	509	1,848	4,093	0.69	30	70	115	0.31
	2016	462	80	105	131	0.50	699	2,830	6,098	0.46	55	90	135	0.65
Perth	2015 (1)	649	43	101	133	0.31	n.d.	n.d.	n.d.	n.d.	n.d.	n.d.	n.d.	n.d.
	2015 (2)	420	52	101	145	0.36	n.d.	n.d.	n.d.	n.d.	n.d.	n.d.	n.d.	n.d.
	2015 (3)	786	54	99	122	0.58	n.d.	n.d.	n.d.	n.d.	n.d.	n.d.	n.d.	n.d.
	2016	629	56	114	152	0.36	n.d.	n.d.	n.d.	n.d.	30	70	120	0.41
Merredin	NI 2016	462	80	111	131	0.52	678	3,297	7,322	0.59	35	70	110	0.35
	IR 2016	462	78	111	131	0.78	521	2,910	4,651	0.73	40	70	115	0.09

Supplemental Table S4: Functional allele diversity between Chevallier and Prior based on significant SNP-associations with phenology.

SNP ID	Gene ID	Gene Name	REF	ALT	Functional Allele	Functional Allele Ref or Alt	Functional Allele Effect direction	Functional allele present in Chevallier?	Functional allele present in Prior?
Chr_1_26220293	HORVU1Hr1G011030		T	C	C	2	-	N	Y
Chr_9_177521890	HORVU0Hr1G030640		G	C	G	1	+	Y	N
Chr_1_26220407	HORVU1Hr1G011030		G	C	G	1	+	Y	N
Chr_2_641454205	HORVU2Hr1G090030		C	T	C	1	+	N	Y
Chr_6_133136483	.		C	T	C	1	-	Y	N
Chr_6_133174007	.		G	A	G	1	-	Y	N
Chr_1_497765114	HORVU1Hr1G072470	LCC	C	T	T	2	-	N	Y
Chr_1_515518660	HORVU1Hr1G076730	GA2ox4	C	T	C	1	-	N	Y
Chr_5_560587533	HORVU5Hr1G080430	CBF10A	G	A	G	1	+	Y	N
Chr_6_262904660	.		T	C	C	2	+	N	Y
Chr_1_515518819	HORVU1Hr1G076730	GA2ox4	G	A	G	1	-	N	Y
Chr_3_548755958	HORVU3Hr1G072810	GA2ox3	G	C	G	1	+	Y	N
Chr_1_441475894	HORVU1Hr1G060810	GID1	T	C	C	2	-	Y	N
Chr_5_463743288	.		G	T	T	2	-	N	Y
Chr_9_73633495	.		C	A	A	2	-	N	Y
Chr_9_73633498	.		G	A	A	2	-	N	Y
Chr_1_514098702	HORVU1Hr1G076420	FT3 (PPD-H2)	A	C	C	2	-	N	Y
Chr_1_515520520	HORVU1Hr1G076730	GA2ox4	A	G	A	1	-	N	Y
Chr_1_515519008	HORVU1Hr1G076730	GA2ox4	A	C	A	1	-	N	Y
Chr_5_565159686	HORVU5Hr1G081620	PRR95	A	G	G	2	-	N	Y
Chr_5_565158282	HORVU5Hr1G081620	PRR95	C	T	T	2	-	N	Y
Chr_5_565156742	HORVU5Hr1G081620	PRR95	A	G	G	2	-	N	Y
Chr_2_29124129	HORVU2Hr1G013400	PPD-H1	G	T	G	1	-	N	Y
Chr_6_38883155	.		G	A	G	1	+	Y	N
Chr_6_43102588	.		C	A	A	2	+	Y	N
Chr_6_70582296	HORVU6Hr1G022330	ZTLb	T	C	C	2	+	Y	N

Chr_6_38883611	.		C	A	C	1	+	Y	N
Chr_6_38883214	.		C	T	C	1	+	Y	N
Chr_2_29126843	HORVU2Hr1G013400	PPD-H1	C	T	C	1	-	N	Y
Chr_2_29124833	HORVU2Hr1G013400	PPD-H1	C	G	C	1	-	Y	N
Chr_2_685788100	HORVU2Hr1G098930	AGL32	C	T	C	1	-	N	Y
Chr_2_523379279	.		C	T	C	1	+	Y	N
Chr_2_523379283	.		T	C	T	1	+	Y	N
Chr_7_49217139	HORVU7Hr1G027560	CO8	G	A	G	1	-	N	Y
Chr_7_37906338	HORVU7Hr1G024000	MADS25-3	C	T	C	1	-	N	Y
Chr_5_586672429	.		G	T	T	2	-	Y	N
Chr_2_637598850	.		G	T	G	1	+	Y	N
Chr_5_463743285	.		C	T	C	1	+	Y	N
Chr_4_6132570	HORVU4Hr1G003060	GRP7a	G	C	C	2	-	N	Y
Chr_1_458877262	HORVU1Hr1G064150	STK	G	A	G	1	+	Y	N
Chr_3_699526755	HORVU3Hr1G117870	GA2betadiox7	A	G	G	2	-	N	Y

Supplemental Table S5: Functional allele diversity between Prior and Clipper based on significant SNP-associations with phenology.

SNP ID	Gene ID	Gene Name	REF	ALT	Functional Allele	Functional Allele Ref or Alt	Functional Allele Effect direction	Functional allele present in Prior?	Functional allele present in Clipper?
Chr_1_26220293	HORVU1Hr1G011030		T	C	C	2	-	Y	N
Chr_9_177521890	HORVU0Hr1G030640		G	C	G	1	+	N	Y
Chr_1_26220407	HORVU1Hr1G011030		G	C	G	1	+	N	Y
Chr_6_133136483	.		C	T	C	1	-	N	Y
Chr_6_133174007	.		G	A	G	1	-	N	Y
Chr_5_560587533	HORVU5Hr1G080430	CBF10A	G	A	G	1	+	N	Y
Chr_6_262904660	.		T	C	C	2	+	Y	N
Chr_3_548755958	HORVU3Hr1G072810	GA2ox3	G	C	G	1	+	N	Y
Chr_1_441475894	HORVU1Hr1G060810	GID1	T	C	C	2	-	N	Y
Chr_2_29124129	HORVU2Hr1G013400	PPD-H1	G	T	G	1	-	Y	N
Chr_6_38883155	.		G	A	G	1	+	N	Y
Chr_6_43102588	.		C	A	A	2	+	N	Y
Chr_6_70582296	HORVU6Hr1G022330	ZTLb	T	C	C	2	+	N	Y
Chr_6_38883611	.		C	A	C	1	+	N	Y
Chr_6_38883214	.		C	T	C	1	+	N	Y
Chr_4_6132570	HORVU4Hr1G003060	GRP7a	G	C	C	2	-	Y	N
Chr_3_699526755	HORVU3Hr1G117870	GA2betadiox7	A	G	G	2	-	Y	N
Chr_5_560732097	HORVU5Hr1G080450	CBF6	G	C	C	2	-	Y	N
Chr_2_29124273	HORVU2Hr1G013400	PPD-H1	G	C	G	1	-	Y	N
Chr_1_458872718	HORVU1Hr1G064150	STK	G	C	C	2	-	N	Y
Chr_1_458874564	HORVU1Hr1G064150	STK	T	G	G	2	-	N	Y
Chr_1_458873507	HORVU1Hr1G064150	STK	A	G	G	2	-	N	Y
Chr_1_458874588	HORVU1Hr1G064150	STK	A	C	C	2	-	N	Y
Chr_1_458874524	HORVU1Hr1G064150	STK	A	C	C	2	-	N	Y
Chr_1_458871879	.		A	T	T	2	-	N	Y

Chr_1_458873355	HORVU1Hr1G064150	STK	T	C	C	2	-	N	Y
Chr_1_458872000	HORVU1Hr1G064150	STK	G	T	T	2	-	N	Y
Chr_1_458875296	HORVU1Hr1G064150	STK	G	T	T	2	-	N	Y
Chr_1_458872807	HORVU1Hr1G064150	STK	A	G	G	2	-	N	Y
Chr_1_458873112	HORVU1Hr1G064150	STK	A	G	G	2	-	N	Y
Chr_1_458873722	HORVU1Hr1G064150	STK	C	T	T	2	-	N	Y
Chr_1_458873946	HORVU1Hr1G064150	STK	A	T	T	2	-	N	Y
Chr_1_458873493	HORVU1Hr1G064150	STK	G	A	A	2	-	N	Y
Chr_1_458876811	HORVU1Hr1G064150	STK	G	C	C	2	-	N	Y
Chr_1_458872569	HORVU1Hr1G064150	STK	C	G	G	2	-	N	Y
Chr_1_458874068	HORVU1Hr1G064150	STK	C	T	T	2	-	N	Y
Chr_5_598563697	HORVU5Hr1G095530	PhyC	C	T	T	2	-	Y	N
Chr_5_598567348	HORVU5Hr1G095530	PhyC	G	T	T	2	-	Y	N
Chr_5_598563278	HORVU5Hr1G095530	PhyC	T	C	C	2	-	Y	N
Chr_5_598561262	HORVU5Hr1G095530	PhyC	T	C	C	2	-	Y	N
Chr_5_598564742	HORVU5Hr1G095530	PhyC	A	G	G	2	-	Y	N
Chr_9_77794179	.		T	C	C	2	-	Y	N
Chr_5_598563899	HORVU5Hr1G095530	PhyC	C	A	A	2	-	Y	N
Chr_5_598566629	HORVU5Hr1G095530	PhyC	A	T	T	2	-	Y	N
Chr_5_598565870	HORVU5Hr1G095530	PhyC	G	A	A	2	-	Y	N
Chr_5_598566192	HORVU5Hr1G095530	PhyC	A	T	T	2	-	Y	N
Chr_5_598566179	HORVU5Hr1G095530	PhyC	G	T	T	2	-	Y	N
Chr_6_72972397	HORVU6Hr1G022770	VEL1	A	T	T	2	+	N	Y
Chr_6_72969719	HORVU6Hr1G022770	VEL1	C	T	T	2	+	N	Y
Chr_6_43102724	.		A	G	A	1	-	Y	N
Chr_5_598567047	HORVU5Hr1G095530	PhyC	C	T	C	1	+	N	Y
Chr_6_38883757	.		A	G	A	1	-	Y	N
Chr_3_653188479	HORVU3Hr1G096410		T	G	T	1	-	Y	N

Supplemental Table S6: Functional allele diversity between Clipper and La Trobe based on significant SNP-associations with phenology.

SNP ID	Gene ID	Gene Name	REF	ALT	Functional Allele	Functional Allele Ref or Alt	Functional Allele Effect direction	Functional allele present in Clipper?	Functional allele present in La Trobe?
Chr_5_560588247	.		G	A	G	1	+	N	Y
Chr_5_560588251	.		G	T	G	1	+	N	Y
Chr_5_560588256	.		A	C	C	2	-	Y	N
Chr_5_560732040	HORVU5Hr1G080450	CBF6	C	T	T	2	-	Y	N
Chr_5_560588191	HORVU5Hr1G080430	CBF10A	T	C	C	2	-	Y	N
Chr_5_560588206	HORVU5Hr1G080430	CBF10A	T	A	A	2	-	Y	N
Chr_2_201118611	HORVU2Hr1G041090	CBF8A	G	A	G	1	-	Y	N
Chr_5_560571147	HORVU5Hr1G080420	CBF3	A	T	A	1	+	N	Y
Chr_5_560587472	HORVU5Hr1G080430	CBF10A	T	C	T	1	+	N	Y
Chr_2_29124273	HORVU2Hr1G013400	PPD-H1	G	C	G	1	-	N	Y
Chr_5_565158226	HORVU5Hr1G081620	PRR95	T	A	A	2	-	Y	N
Chr_1_458877362	.		G	A	A	2	-	N	Y
Chr_5_565158282	HORVU5Hr1G081620	PRR95	C	T	T	2	-	Y	N
Chr_5_565159686	HORVU5Hr1G081620	PRR95	A	G	G	2	-	Y	N
Chr_5_565156742	HORVU5Hr1G081620	PRR95	A	G	G	2	-	Y	N
Chr_7_238908679	.		A	G	G	2	-	N	Y
Chr_2_29124129	HORVU2Hr1G013400	PPD-H1	G	T	G	1	-	N	Y
Chr_6_38883611	.		C	A	C	1	+	Y	N
Chr_6_38883155	.		G	A	G	1	+	Y	N
Chr_7_298065848	.		A	G	G	2	-	N	Y
Chr_6_38883214	.		C	T	C	1	+	Y	N
Chr_6_43102588	.		C	A	A	2	+	Y	N
Chr_6_70582296	HORVU6Hr1G022330	ZTLb	T	C	C	2	+	Y	N
Chr_6_72972397	HORVU6Hr1G022770	VEL1	A	T	T	2	+	Y	N
Chr_6_70578423	HORVU6Hr1G022330	ZTLb	T	A	A	2	+	Y	N

Chr_6_70577264	HORVU6Hr1G022330	ZTLb	A	G	G	2	+	Y	N
Chr_6_70580833	HORVU6Hr1G022330	ZTLb	G	A	A	2	+	Y	N
Chr_6_70579360	HORVU6Hr1G022330	ZTLb	G	A	A	2	+	Y	N
Chr_6_72969719	HORVU6Hr1G022770	VEL1	C	T	T	2	+	Y	N
Chr_5_598563697	HORVU5Hr1G095530	PhyC	C	T	T	2	-	N	Y
Chr_5_598563278	HORVU5Hr1G095530	PhyC	T	C	C	2	-	N	Y
Chr_5_598561262	HORVU5Hr1G095530	PhyC	T	C	C	2	-	N	Y
Chr_5_598564742	HORVU5Hr1G095530	PhyC	A	G	G	2	-	N	Y
Chr_9_77794179	.		T	C	C	2	-	N	Y
Chr_5_598567348	HORVU5Hr1G095530	PhyC	G	T	T	2	-	N	Y
Chr_5_598563899	HORVU5Hr1G095530	PhyC	C	A	A	2	-	N	Y
Chr_5_598566629	HORVU5Hr1G095530	PhyC	A	T	T	2	-	N	Y
Chr_5_598566192	HORVU5Hr1G095530	PhyC	A	T	T	2	-	N	Y
Chr_5_598566179	HORVU5Hr1G095530	PhyC	G	T	T	2	-	N	Y
Chr_5_598565870	HORVU5Hr1G095530	PhyC	G	A	A	2	-	N	Y
Chr_6_43102724	.		A	G	A	1	-	N	Y
Chr_5_598567047	HORVU5Hr1G095530	PhyC	C	T	C	1	+	Y	N
Chr_2_685788100	HORVU2Hr1G098930	AGL32	C	T	C	1	-	Y	N
Chr_6_38883757	.		A	G	A	1	-	N	Y
Chr_2_128270425	HORVU2Hr1G032710	GW7	A	G	G	2	-	N	Y
Chr_7_49217139	HORVU7Hr1G027560	CO8	G	A	G	1	-	Y	N
Chr_2_128270958	HORVU2Hr1G032710	GW7	T	A	A	2	-	N	Y
Chr_6_70578602	HORVU6Hr1G022330	ZTLb	A	G	A	1	-	N	Y
Chr_9_12161515	HORVU0Hr1G003020	BM3	A	G	G	2	-	N	Y
Chr_9_12159779	HORVU0Hr1G003020	BM3	G	A	A	2	-	N	Y
Chr_9_12159195	HORVU0Hr1G003020	BM3	C	T	T	2	-	N	Y
Chr_9_12158311	HORVU0Hr1G003020	BM3	A	T	T	2	-	N	Y
Chr_6_70580335	HORVU6Hr1G022330	ZTLb	T	C	T	1	-	N	Y
Chr_6_70580834	HORVU6Hr1G022330	ZTLb	T	C	T	1	-	N	Y
Chr_6_70577373	HORVU6Hr1G022330	ZTLb	T	C	T	1	-	N	Y
Chr_6_70580571	HORVU6Hr1G022330	ZTLb	A	G	A	1	-	N	Y

Chr_6_70578044	HORVU6Hr1G022330	ZTLb	A	G	A	1	-	N	Y
Chr_9_12159591	HORVU0Hr1G003020	BM3	C	T	T	2	-	N	Y
Chr_5_560570288	HORVU5Hr1G080420	CBF3	A	G	A	1	+	N	Y
Chr_9_12161328	HORVU0Hr1G003020	BM3	A	C	C	2	-	N	Y
Chr_6_504463075	HORVU6Hr1G072620	CO2	C	T	T	2	-	N	Y
Chr_5_560588278	.		G	A	G	1	+	N	Y
Chr_6_504461160	HORVU6Hr1G072620	CO2	G	A	A	2	-	N	Y
Chr_6_504460853	HORVU6Hr1G072620	CO2	A	G	G	2	-	N	Y
Chr_6_139882589	HORVU6Hr1G032220	TT16	G	A	A	2	-	N	Y
Chr_5_586672429	.		G	T	T	2	-	N	Y

Supplemental Table S7: Functional allele diversity between Chevallier and Prior based on significant SNP-associations with grain yield.

SNP ID	Gene ID	Gene Name	REF	ALT	Functional Allele	Functional Allele Ref or Alt	Functional Allele Effect direction	Functional allele present in Chevallier?	Functional allele present in Prior?
Chr_1_26220293	HORVU1Hr1G011030		T	C	C	2	-	N	Y
Chr_5_565156748	HORVU5Hr1G081620	PRR95	G	C	C	2	-	N	Y
Chr_5_565157545	HORVU5Hr1G081620	PRR95	C	G	G	2	-	N	Y

Supplemental Table S8: Functional allele diversity between Prior and Clipper based on significant SNP-associations with grain yield.

SNP ID	Gene ID	Gene Name	REF	ALT	Functional Allele	Functional Allele Ref or Alt	Functional Allele Effect direction	Functional allele present in Prior?	Functional allele present in Clipper?
Chr_1_26220293	HORVU1Hr1G011030		T	C	C	2	-	Y	N
Chr_5_560732097	HORVU5Hr1G080450	CBF6	G	C	C	2	-	Y	N
Chr_3_117876749	HORVU3Hr1G027460	CKX	C	T	C	1	-	Y	N
Chr_3_198828628	HORVU3Hr1G035680	FPF1	C	T	C	1	-	Y	N

Supplemental Table S9: Functional allele diversity between Prior and Clipper based on significant SNP-associations with plant height.

SNP ID	Gene ID	Gene Name	REF	ALT	Functional Allele	Functional Allele Ref or Alt	Functional Allele Effect direction	Functional allele present in Prior?	Functional allele present in Clipper?
Chr_3_634079937	HORVU3Hr1G090980	GA20ox2	C	T	T	2	-	N	Y

Supplemental Table S10: Functional allele diversity between Clipper and La Trobe based on significant SNP-associations with plant height.

SNP ID	Gene ID	Gene Name	REF	ALT	Functional Allele	Functional Allele Ref or Alt	Functional Allele Effect direction	Functional allele present in Clipper?	Functional allele present in La Trobe?
Chr_5_560570638	HORVU5Hr1G080420	CBF3	G	C	C	2	-	Y	N
