# Supplementary material for: Hybridisation‐based target enrichment of phenology genes to dissect the genetic basis of yield and adaptation in barley
Source: Plant Biotechnol J. 2018 Dec 6;17(5):932–44. doi: 10.1111/pbi.13029 (PMC6587706; doi:10.1111/pbi.13029)
Supplement: Supplementary file 3 — Appendix S1. Detailed description of additional experimental procedures. [file PBI-17-932-s007.docx]

**Supporting Experimental Procedures**

**Targeted resequencing of phenology genes**

The targeted sequences comprised genes related to phenology and the development of meristem and inflorescences. Additionally, it contained a selection of genes related to grain yield and quality traits. Scientific literature was comprehensively mined for genes implicated in the aforementioned processes and the corresponding nucleotide sequences were extracted from NCBI GenBank. In addition, barley ortholog of flowering genes from the other grass species, such as bread wheat and rice, were identified through BLASTN search (e-value <1e^-10^) and included. Complete genomic DNA sequences were selected were possible. Where absent, cDNA and partial cDNA sequences were included instead.

Targeted enrichment of genomic DNA regions was performed by solution-based hybrid capture using a synthetic library consisting of 13,588 probes (MYbaits, MYcroarray^®^, Ann Arbour, MI, USA) following the manufacturer’s protocol version 2.3.1. The capture oligonucleotides were 80 nt long and were designed to target approximately 600 kb of repeat- and organelle-masked genic sequence (190 phenology-related genes, derived from *Hordeum vulgare* Genome assembly 082214v1) with a 2x tiling density. For probe design, the sequence of the gene was repeat-masked based on organelle, transposable elements (TREP), and high copy number in the barley reference genome (genome assembly 082214v1). Hybridization of the customised RNA baits with sample library pools was performed at 65°C for 24 hours. Amplification of the resulting bead-bound enriched libraries was performed using KAPA HiFi HotStart Ready Mix and adapter-specific primers. The concentration of each enriched sample library was determined using a Qubit fluorometer (Thermo Fisher Scientific), and the library size with Tapestation (Agilent) for subsequent library titration and next-generation sequencing. Libraries were titrated using the KAPA Library Quantification Kit. Each pooled library was sequenced on an Illumina HiSeq3000 instrument to generate about 0.5 million 2x150 bp paired-end reads per sample. Genome sequencing was conducted at AgriBio, (Centre for AgriBioscience, Bundoora, VIC).

**Sequence alignment, variant discovery, genotype calling, and variant prediction**

Fastq sequence files were post-run filtered using Nuclear software v3.6.16 (GYDLE Inc., Montreal, Canada) based on a minimum nucleotide q-score of 20 and a minimum read length of 50 bp. The resulting high-quality (HQ) reads were aligned to the target molecules, which included 5 kb flanking regions on either side of the genes used for RNA probe design, using Nuclear with low stringency high-scoring segment pairs (HSPs) with minimum read length 30 bp, with sensitivity 13 (consecutive identities), and up to 3 mismatches per window of 30 bases (90% identity). Reads aligning to the genomic regions targeted in the capture assay were retained as on-target reads. These on-target reads were re-aligned using Nuclear software with higher stringency (HSPs with minimum read length 50 bp, sensitivity 25 and maximum of 3 mismatches per window of 50 bases) to the latest release of the barley reference genome assembly (IBSC v2; Mascher et al., 2017).

The capture assay was designed to capture 600 kb of target sequence which corresponded to 1.9 Mbp of target gene space in the current barley genome assembly IBSC v2 (Mascher et al., 2017). SNP variant discovery and genotype calling was performed using custom Perl scripts to produce a VCF version 4.2 genotype file based on the alignment files. To eliminate any possibility for ascertainment biased that can arise when SNP discovery is performed using only a subset of samples, the entire population was selected to discover SNPs. We included only the SNP loci that were covered by at least four reads. Variant effect prediction of SNP captured within gene exon (coding) sequence was performed using the Ensembl Variant Effect predictor toolset (Ensembl Variant Effect Predictor web interface http://www.ensembl.org/vep). Only the SNPs with <10% missing values and a minor allele frequency >1% were used for subsequent analyses.

### **Population structure and genotypic data analysis**

Polymorphic information content (PIC), an index for evaluating the frequency of nucleotide variant occurrence in a population, was calculated for each SNP using the formula

$PIC=1-\sum_{i=1}^{l} P_{i}^{2}-\sum_{i=1}^{l-1} \sum_{j=i+1}^{l} 2P_{i}^{2}P_{j}^{2}$ (1)

where *P_i_* and *P_j_* are the population frequency of the *i*th and *j*th allele (Nagy et al, 2012). Major allele frequency, minor allele frequency (MAF), gene diversity and genetic distance based on IBS (identity-by-state similarity) were calculated and a NJ (Neighbor-Joining) dendrogram was computed, all using TASSEL v.5.2.39 software (Bradbury et al., 2007). The final set of SNP used for subsequent analyses was filtered to contain those with a MAF above 1% and genotype call rate above 10%.

The model-based clustering algorithm of ADMIXTURE v.1.3.0 was used to investigate subpopulation structure of the barley diversity panel. A preliminary analysis was performed in 100 replicate runs by inputting successive values of K from 1 to 18. A 10-fold cross validation (CV) procedure with penalised estimation (lambda=250, e=0.05) was performed with 100 different fixed initial seeds for each K-value. The most likely K-value was determined using ADMIXTURE’s CV error values. The software CLUMPP (Jakobsson and Rosenberg, 2007) v.1.1.2 was used to obtain the optimal alignments of 100 replicates for each K-value. Individual genotype membership proportions were averaged across runs according to the permutation with the greatest symmetric similarity coefficient. The output from CLUMPP for the optimal K was used to make plots using the cluster visualization program Pophelper v.2.2.3 (http://royfrancis.github.io/pophelper/) implemented in R software (http://www.R-project.org/).

Principal component analysis (PCA) was also conducted based on all markers data using TASSEL v.5.2.39 to summarize the genetic structure and variation present in the barley germplasm. The first three principal components were plotted against each other using ‘scatter plot’ function in Microsoft Excel 2016. A neighbour-joining (NJ) tree was constructed based on genetic distances calculated in TASSEL v.5.2.39. The substructure in the collection using different methodologies was compared and the final *K* value using ADMIXTURE was ascertained.

**Linkage disequilibrium and haplotype analysis**

Genome-wide LD analysis was performed among the panel and subgroups by pair wise comparisons among the intra-chromosomal SNP markers using Haploview (Barrett et al., 2005) v.4.2. LD was estimated by using squared allele frequency correlations (*r*^2^) between the intra-chromosomal pairs of loci (Weir, 1996). The loci were considered to be in significant LD when *P* < 0.001. The pattern and distribution of intra-chromosomal LD was visualized and studied from LD plots generated for each chromosome by Haploview. To investigate the average LD decay in the the panel, significant intra-chromosomal *r*^2^ values were plotted against the physical distance (Mb) between markers by a sliding window approach with 100 Mb windows.

**Association analysis**

Genome wide association studies were performed using TASSEL v.5.2.39 software (Bradbury et al., 2007) using SNP with <10% missing values and minor allele frequency >1%. Different statistical models were used to calculate P-values for putative marker-trait associations which included population structure (Q) and the kinship matrix (K) to account for population structure to avoid spurious associations.

We followed the formula

$Y=X\beta+Zu+e$, (2)

where Y is the vector of observations; β is an unknown vector containing fixed effects, including genetic marker and population structure (Q); u is an unknown vector of random additive genetic effects from multiple background QTL for individuals/lines; *X* is the top five principal components (PCs) from PCA, explaining 30% of the cumulative variance of all markers; *Z* is an identity matrix; and e is the unobserved vector of random residual.

The following models were tested: i) Naïve model: GLM without any correction for population structure; ii) P-model: GLM with PCs as a correction for population structure; iii) Q+K model: Compressed Mixed Linear Model (MLM) with population structure (Q) matrix (PCs) and kinship (K) matrix (matrix of genetic similarity based on simple SNP matching coefficients, which was also used for constructing the neighbour-joining tree) as a correction for population structure. Results were compared, and according to the quantile-quantile (Q-Q) plot, the MLM model incorporating Q and K was suitable for this study.

For the MLM model and analyses, multiple testing was performed to assess the significance of marker trait associations using the qvalue (Storey, 2002) v.2.8.0 R package (R 3.4.2) employing the smoother method (Storey and Tibshirani, 2003), an extension of the false discovery rate (FDR) method. Lambda was selected as 0 which estimates πi(0)=1, which produces a list of significant tests equivalent to the Benjamini and Hochberg (1995) procedure and is considered a conservative case of the qvalue methodology. Only markers with qFDR < 0.05 were considered to be significant. Q-values were calculated using the R-package q-value. The Manhattan plot was drawn with qman (Turner, 2018) v.0.1.4.

 The proportion of genetic variance over the total variance is defined as heritability (h^2^):

$h^{2}=\frac{\sigma_{a}^{2}}{\sigma_{a}^{2}+ \sigma_{e}^{2}}$ (3)

**REFERENCES**

Barrett JC, Fry B, Maller J, Daly MJ. Haploview: analysis and visualization of LD and haplotype maps. Bioinformatics. 2005;21:263-265.

Benjamini Y, Hochberg Y. Controlling the false discovery rate: a practical and powerful approach to multiple testing. JR Stat Soc Series B Stat Methodol. 1995;57:289-300.

Bradbury PJ, Zhang Z, Kroon DE, Casstevens TM, Ramdoss Y, Buckler ES. TASSEL: software for association mapping of complex traits in diverse samples. Bioinformatics. 2007;23:2633-2007.

Jakobsson M, Rosenberg NA. CLUMPP: a cluster matching and permutation program for dealing with label switching and multimodality in analysis of population structure.

Bioinformatics. 2007;23:1801-1806.

Mascher M, Gundlach H, Himmelbach A, Beier S, Twardziok SO, Wicker T, et al. A chromosome conformation capture ordered sequence of the barley genome. Nature. 2017;544:427-433.

Nagy S, Poczai P, Cernák I, Gorji AM, Hegedűs G, Taller J. PICcalc: an online program to calculate polymorphic information content for molecular genetic studies. Biochem. Genet. 2012;50:670-672.

Storey, JD. A direct approach to false discovery rates. JR Stat Soc Series B Stat Methodol. 2002;64:479-98.

Storey JD, Tibshirani R. Statistical significance for genomewide studies. Proc Natl Acad Sci USA. 2003;100:9440-9445.

Turner SD. qqman: an R package for visualizing GWAS results using Q-Q and manhattan plots. J Open Source S. 2018;3:731

Weir BS. Genetic Data Analysis II: Methods for Discrete Population Genetic Data. 2nd ed. Massachusetts: Sinauer Associates; 1996.
